# Harmonizing the National Lung Screening Trial: Kernel resolution and field of view compensation

**DOI:** 10.1371/journal.pone.0352075

**Published:** 2026-10-05

**Authors:** Aravind R. Krishnan, Michael E. Kim, Chenyu Gao, Gaurav Rudravaram, Trent Schwartz, Andre T. S. Hucke, Lucas W. Remedios, Thomas Z. Li, Kaiwen Xu, Kim L. Sandler, Yency Forero Martinez, Sanja Antic, Fabien Maldonado, Lianrui Zuo, Bennett A. Landman

**Affiliations:** 1 Department of Electrical and Computer Engineering, Vanderbilt University, Nashville, Tennessee, United States of America; 2 Department of Computer Science, Vanderbilt University, Nashville, Tennessee, United States of America; 3 Department of Biomedical Engineering, Vanderbilt University, Nashville, Tennessee, United States of America; 4 Insitro, South San Francisco, California, United States of America; 5 Department of Radiology and Radiological Sciences, Vanderbilt University Medical Center, Nashville, Tennessee, United States of America; 6 Division of Allergy, Pulmonary and Critical Care Medicine, Department of Medicine, Vanderbilt University Medical Center, Nashville, Tennessee, United States of America; 7 Department of Medicine, Vanderbilt University Medical Center, Nashville, Tennessee, United States of America; 8 Department of Thoracic Surgery, Vanderbilt University Medical Center, Nashville, Tennessee, United States of America; 9 Vanderbilt University Institute of Imaging Science, Vanderbilt University Medical Center, Nashville, Tennessee, United States of America; University of Pisa, ITALY

## Abstract

Low-dose chest computed tomography scans acquired in multi-centre studies exhibit heterogeneity in the form of varying spatial resolution, reconstruction kernel and semantic field-of-view that hinder the reproducibility of quantitative measures and generalizability of downstream analyses. The National Lung Screening Trial (NLST) provides a large, open collection of low-dose chest CT scans characterized by heterogeneous spatial resolution, reconstruction kernels and field-of-view configurations. This diversity presents an opportunity to standardize the data, enabling reproducible biomarker estimation and broader reuse of the NLST as a homogeneous quantitative imaging resource. In this work, we present a standardization pipeline that was applied to 105,191 scans from 23,669 participants in the NLST. The pipeline addresses the following elements: (1) Resampling through-axial-plane (z) spatial resolution to 1.0 mm (2) Harmonization of reconstruction kernels across intra-vendor (paired) and inter-vendor (unpaired) settings using an anatomy guided multipath cycleGAN where scans were mapped to a reference Siemens B30f (soft) and Siemens B50f (hard) kernel (3) Semantic field-of-view extension across all scans. Quantitative consistency across percent emphysema and body composition assessment (skeletal muscle and subcutaneous adipose tissue volumes) was evaluated in paired, cross-sectional and longitudinal settings. For paired data, harmonization improved agreement of quantitative measures relative to the soft kernel reference. Cross-sectional analyses demonstrated reduced inter-kernel variability, with small to medium effect sizes (Cohen’s *d* = 0.2 to 0.5) for percent emphysema and improved correlations with anthropometric measures post field-of-view extension. Longitudinal analysis using linear mixed effect models, showed reduced kernel bias and improved longitudinal consistency of biomarkers reflected by the increase in intraclass correlation coefficients. This work provides a harmonized NLST imaging resource that enables consistent and reliable quantitative biomarker estimation for lung cancer screening, with open source code released at https://github.com/MASILab/Harmonized_NLST_dataset and the harmonized dataset to be made publicly available once data return to the NLST is completed.

## 1 Introduction

Lung cancer remains the leading cause of cancer-related mortality and low-dose chest computed tomography (LDCT) has emerged as an effective screening tool for high-risk populations by enabling early detection of lung cancer nodules [1,2]. The National Lung Screening Trial (NLST), a landmark randomized controlled trial of 53,454 participants, assigned individuals to undergo three annual screenings with either LDCT or chest radiography, demonstrating a 20% decrease in lung cancer mortality in the LDCT group as compared to radiography [3,4]. Following its completion, the NLST imaging dataset was made publicly available [5], providing a large-scale longitudinal LDCT cohort that has substantially advanced lung cancer risk modelling and quantitative imaging research. Researchers have leveraged the NLST to develop prediction models for future lung cancer risk from a single timepoint CT scan [6], longitudinal chest CTs [7] and a combination of imaging and clinical records [8,9]. Furthermore, the NLST has enabled quantitative imaging studies to study associations between lung cancer risk and imaging biomarkers that include emphysema [10], coronary artery calcification [11] and body composition [12].

Despite its transformative impact and widespread adoption in quantitative imaging research, the full potential of the NLST dataset has yet to be realized. A major but often overlooked challenge is the substantial heterogeneity in image acquisition protocols across the cohort. The NLST comprises LDCT scans acquired at 33 institutions using 23 distinct scanner models and 41,019 unique acquisition parameter combinations, as encoded by the DICOM Series Description. Consequently, the dataset exhibits considerable variability in through-plane spatial resolution (slice thickness), reconstruction kernels, and field of view (FOV) (Fig 1). Such acquisition variability directly affects quantitative imaging measurements and limits reproducibility across studies. Differences in slice thickness introduce partial volume effects, with thicker slices showing increased spatial averaging [13]. These effects have been shown to impact CT-derived radiomic features of lung nodules [14,15] and attenuation based measures that include percent emphysema [16]. Similarly, reconstruction kernels influence image texture, noise characteristics and edge definition [17], affecting emphysema [18] and body composition measures [19].

**Fig 1:**
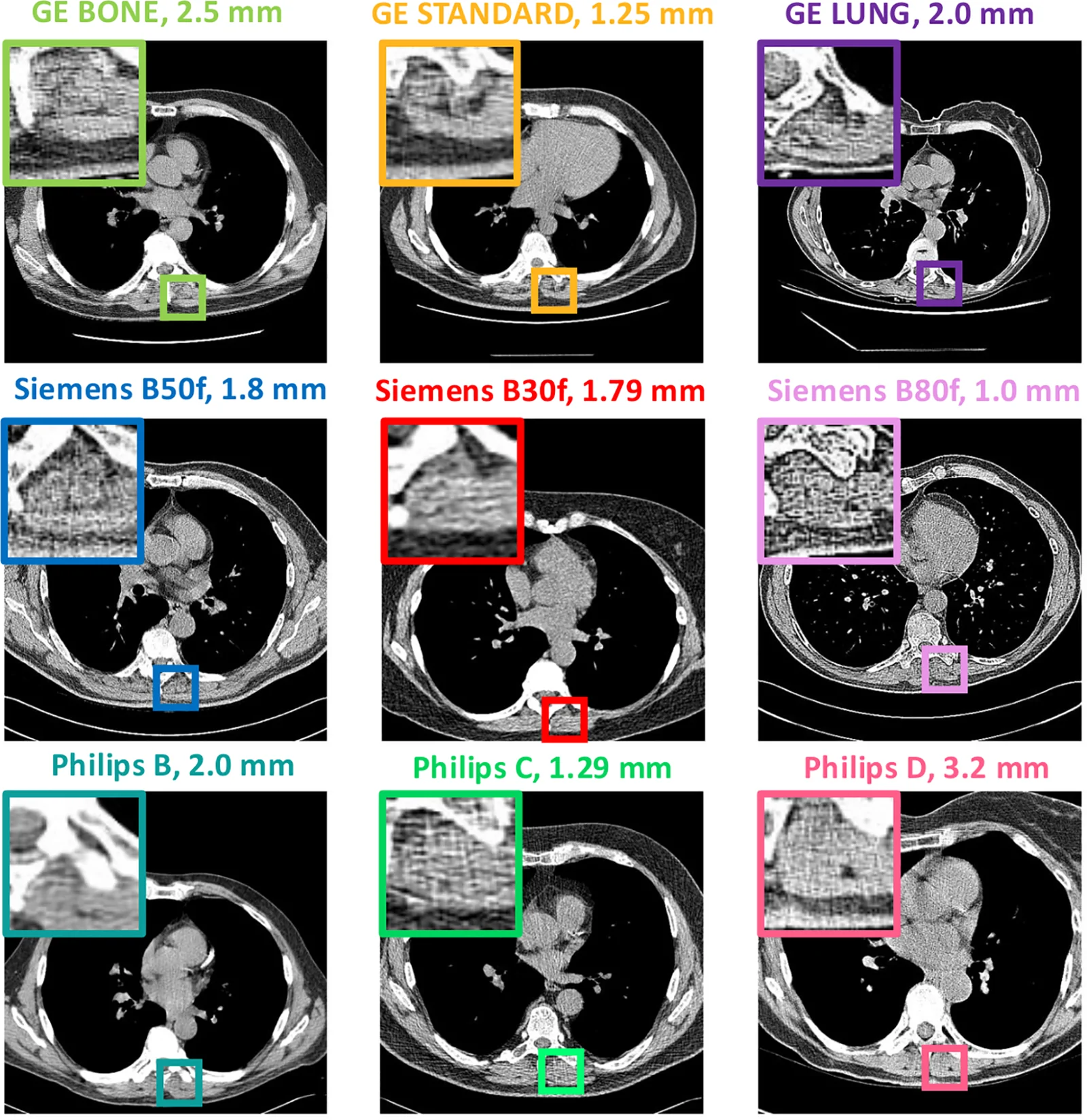
Acquisition heterogeneity in scans obtained from different vendors in the NLST. The National Lung Screening Trial houses heterogeneous low-dose chest CT scans acquired from multiple institutions and vendors. Image variability arises from differences in through-plane spatial resolution, reconstruction kernels and semantic field-of-view. Reconstruction kernels influence the pixel noise and image texture in the underlying anatomy, creating differences in quantitative biomarker measures. In addition, scanner specific field-of-view configurations may truncate anatomy, affecting body composition assessment. These sources of heterogeneity motivate the need for a standardized dataset that enables consistent and reproducible quantitative image measures.

Lung cancer screening protocols often restrict the field of view, resulting in scanner dependent truncation patterns [20] that obscure skeletal muscle and subcutaneous adipose tissue (SAT). Such truncation leads to incomplete body composition quantification and limits the value of LDCTs in opportunistic comprehensive assessment [20]. Prior studies have demonstrated that harmonization strategies can improve the robustness and reproducibility of quantitative imaging biomarkers across heterogeneous CT acquisitions [21], while FOV standardization or extension [20] approaches can recover anatomically consistent representations for downstream analysis. Together, these observations highlight the need for systematic harmonization of acquisition-related variability to ensure consistent and reproducible quantitative biomarkers across the NLST.

While variability in slice thickness, reconstruction kernels and FOV can be addressed individually through resampling, kernel harmonization [21–23] and semantic FOV extension [20] respectively, comprehensive standardization of the NLST requires their integration into a unified framework. To address this need, we propose a multi-stage standardization pipeline that performs through-plane resampling, multi-domain reconstruction kernel harmonization and semantic FOV extension across all scans and timepoints in the NLST. Through plane resampling standardizes images to a slice thickness of 1 mm, mitigating slice thickness variability across scans, which otherwise introduces variability in quantitative measures. Following resampling, nine representative kernels obtained from three distinct scanners in the NLST are harmonized using an anatomically guided multipath cycleGAN model [23], addressing intra-scanner and inter-scanner harmonization. For intra-scanner pairs with a one-to-one correspondence, hard kernels are mapped to their corresponding soft kernel, while inter-scanner harmonization translates all kernels to reference styles defined by Siemens B30f (soft kernel) and Siemens B50f (hard kernel). We choose the Siemens kernels as references for inter scanner harmonization due to their impact on emphysema quantification in lung CT [16,24]. Harmonized images subsequently undergo semantic FOV extension [25] to restore truncated skeletal muscle and SAT, enabling complete body composition assessment. We evaluate the effectiveness of standardization using percent emphysema and body composition measures, derived from anatomical labels generated by TotalSegmentator [26]. Performance is assessed at three levels: a) intra-scanner paired analysis b) cross-sectional comparisons at each timepoint and c) longitudinal evaluation of biomarker stability within subjects. The resulting standardized NLST dataset enables consistent and reproducible quantitative image analysis across multiple scanners and timepoints, thereby expanding its utility for the research community.

## 2 Materials and methods

### 2.1 NLST population

The NLST is a prospective randomized control screening trial where participants were randomized to obtain a low-dose chest CT (LDCT) or a chest radiograph [3]. Participants included in this study were between the ages of 55 and 74 years, with a smoking history of 30 packs years (current smokers) or had quit within the last 15 years (former smokers). Participants were screened at three different timepoints (T0, T1 and T2) at one year intervals with the first screening performed at timepoint T0, immediately after patient randomization. Subjects diagnosed with lung cancer were not offered follow up scans. The primary endpoint of the NLST was lung cancer mortality.

### 2.2 Data curation for standardization

We standardize the CT arm of the NLST (dataset version: **2011.02.03/04.07.22, ClinicalTrials.gov identifier NCT00047385**) comprising of LDCT scans reconstructed with different acquisition parameters from multiple vendors. We obtained all data upon request with a data use agreement (https://cdas.cancer.gov/learn/nlst/images/). All DICOM (Digital Imaging and Communications in Medicine) scans are converted to NIfTI (Neuroimaging Informatics Technology Initiative) using the dcm2niix tool (version 1.0.2) [27], leading to 25206 participants with 195373 scans. The dataset consists of scans with variable slice thickness, slice spacing and 39 distinct reconstruction kernels from four different vendors. We apply an exclusion criteria as a first round of filtering (Fig 2) that excludes images with missing kernel information, scans from the Toshiba vendor, non-standard image orientations, excessive slice thickness (≥ 4 mm) or slice spacing (≥ 4 mm) in the axial plane, four dimensional CT volumes and underrepresented kernels at baseline. Scans reconstructed from the Toshiba vendor exhibit a different noise texture when compared to Siemens, GE and Philips [28]. This makes it particularly challenging to harmonize Toshiba kernels to a common reference [29], resulting in their exclusion. We defined a reconstruction kernel as underrepresented if the number of scans reconstructed with said kernel is less than one hundred at baseline, as baseline contained the greatest diversity of kernels. We provide a list of excluded kernels across all timepoints in the supplementary material (**Supplementary table ST1** in S1 File). After applying the filtering criteria, 105,191 scans from 23669 participants remained. These scans were reconstructed using the following nine representative kernels from three vendors: Siemens (B50f, B30f, B80f), GE (BONE, LUNG, STANDARD) and Philips (B,C,D).

**Fig 2:**
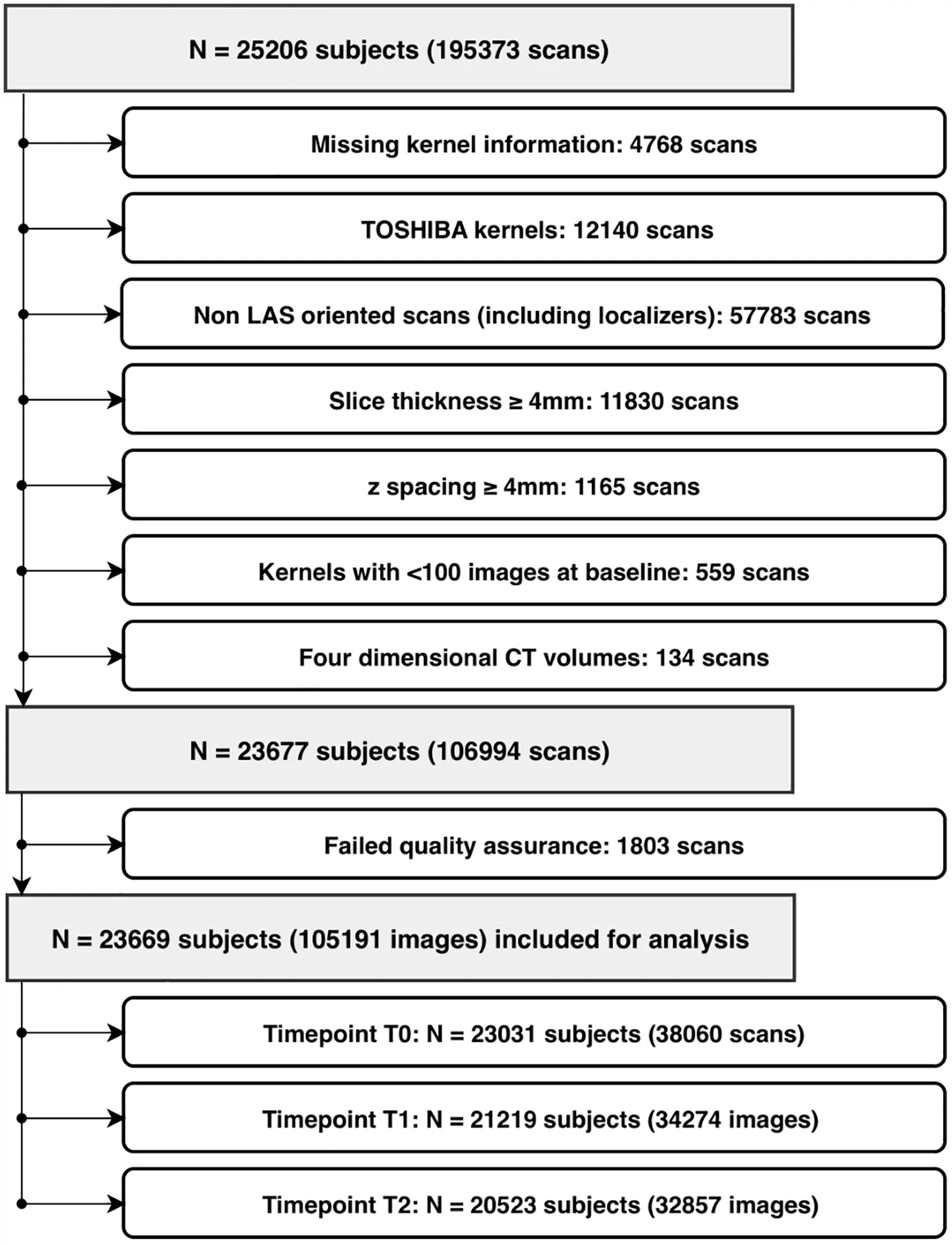
Inclusion and exclusion criteria of images in the NLST. The inclusion criteria for the NLST dataset standardization begins with 25206 subjects having 195373 scans. Scans were excluded based on metadata information, reconstruction characteristics, image orientation, spatial resolution, slice thickness, dimensionality and manual quality assurance. A total of 23669 subjects with 105,191 scans were available for analysis across three different timepoints, reconstructed using nine representative kernels from Siemens, GE and Philips vendors.

We conducted a second round of quality assurance through manual visual assessment to ensure that scans represented a chest CT. We used the quality control tool developed by Kim et al.[30] to review triplanar views of each scan, classifying them as “yes”, “no” or “maybe” based on image quality. 1803 scans that were labelled as no or maybe were excluded. Common reasons for exclusion included incomplete CT volumes with missing slices, incomplete lung coverage in the display field of view (missing apical or basal lung regions), motion artifacts, truncation artifacts producing blank or corrupted slices, shadow artifacts in dual phase acquisitions, stair step artifacts and reconstruction failures. Rare cases of exclusion involved cupping artifacts, irregular lung anatomy, metal artifacts and image distortions (ruptured breast implants) (S1 Fig).

### 2.3 NLST standardization pipeline

To reduce acquisition related heterogeneity in the NLST, we implemented a multi-stage standardization pipeline comprising of spatial resampling in the through-plane, reconstruction kernel harmonization using an anatomy guided multipath cycleGAN and semantic field of view extension to impute missing skeletal muscle and SAT (Fig 3). These stages address variability in through-axial-plane resolution, reconstruction kernels and truncated FOVs that impact quantitative measurements. We evaluate the efficacy of standardization using biomarker measurements derived from the images that include percent emphysema and body composition measures derived from skeletal muscle and SAT.

**Fig 3:**
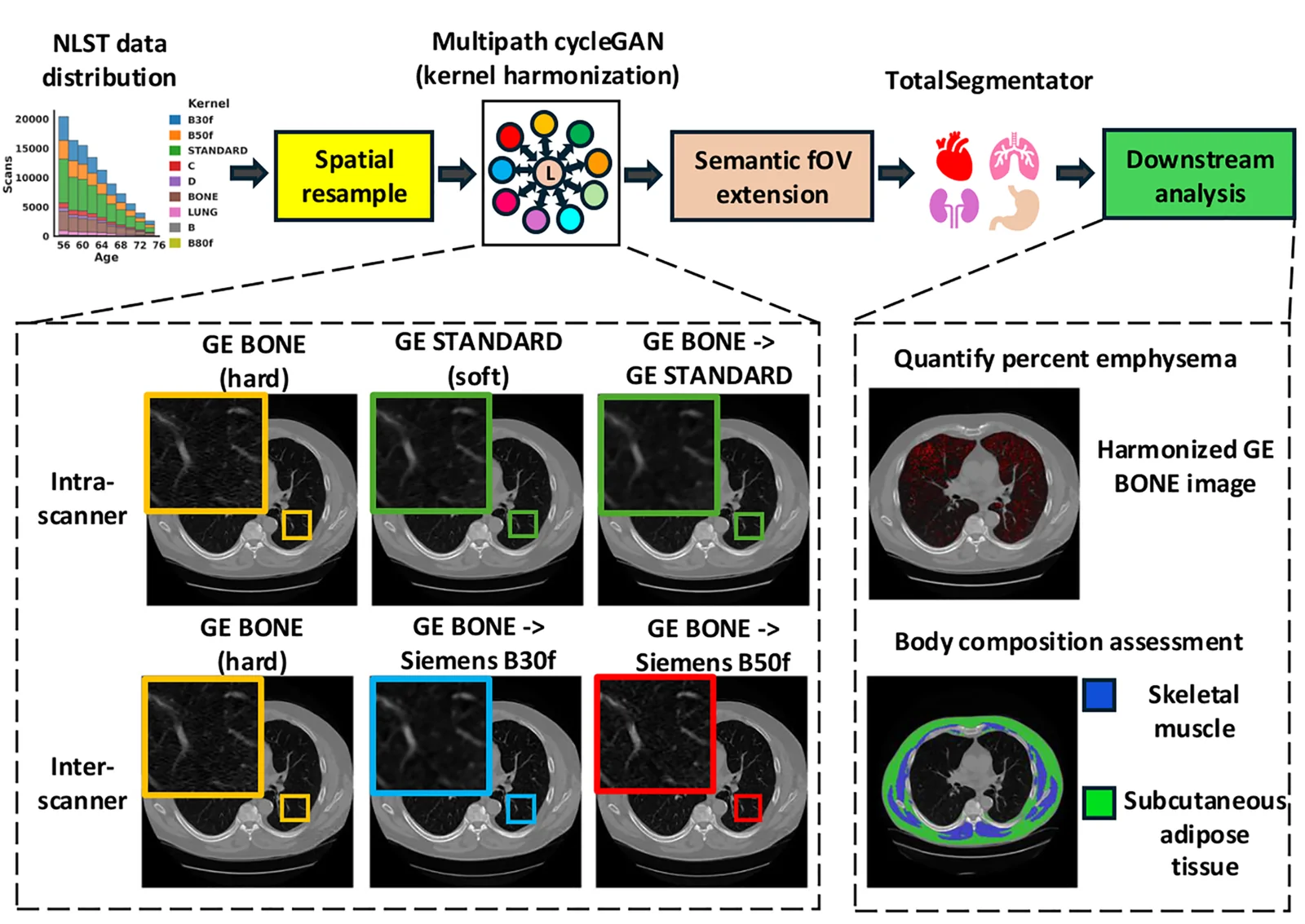
Proposed standardization pipeline to address acquisition heterogeneity. Heterogeneous NLST scans are resampled to a through-plane resolution of 1.0 mm while preserving the in-plane resolution. Reconstruction kernels are harmonized using a multipath cycleGAN with a shared latent space “L”, enabling multi-domain harmonization within and across scanners. The harmonized images undergo semantic field of view extension, where muscle and subcutaneous adipose tissue are out painted beyond truncation. Anatomical masks are subsequently derived using TotalSegmentator, which are used for downstream analysis to obtain percent emphysema and body composition measures.

#### 2.3.1 Spatial resampling and image preprocessing.

The through-plane spatial resolution of the NLST scans varied across images and can introduce partial volume effects that impact downstream analysis. Therefore, we resampled all images to have a through-axial-plane resolution of 1.0 mm using cubic spline interpolation implemented in *nibabel* (version 5.3.2). The native in-plane resolution was preserved across all images. The in-plane display field-of-view (DFOV) differed across images obtained from Siemens, GE and Philips vendors. The mismatch in DFOV during cross vendor harmonization can result in artifacts beyond the FOV extent in images with circular DFOVs [31]. Therefore, we standardized the DFOV to be circular across all the scans before training the harmonization model [21]. All images were clipped to a range of [−1024, 3072] Hounsfield units (HU) to ensure consistent intensities [22].

#### 2.3.2 Training data for kernel harmonization.

Within a given vendor in the NLST, subjects can have an image reconstructed with a hard kernel and a corresponding soft kernel, with a one-to-one correspondence existing between them. Such image pairs are “paired” kernels, obtained from the same projection data. We obtain kernel pairs by matching scanner acquisition parameters that include peak kilovoltage, reconstruction diameter, exposure time, x-ray tube current, exposure and spiral pitch factor. All pairs with mismatched meta data were excluded. Although scans were reconstructed from the same projection data, scan pairs exhibit subtle positional differences within the FOV. Therefore, we conducted a two-step quality control process involving: 1) manual inspection of the difference image to ensure alignment and 2) quantitative evaluation using root mean squared error (RMSE).

We obtain a total of six different reconstruction kernel pairs at each timepoint: Siemens (B30f, B50f), Siemens (B30f, B80f), GE (BONE, STANDARD), GE (LUNG, STANDARD), Philips (B, D), and Philips (C, D). A detailed list of the number of pairs at each timepoint is provided in Table 1. While kernels from the same vendor enabled paired kernel harmonization, all other possible combinations of kernels across vendors served as data for cross vendor harmonization. We only considered the pairs at timepoint T0 to train our kernel harmonization model, where the data was split into 70% for training, 15% for validation and 15% for testing provided (see Table 2 for sample size splits). In addition to the testing data from timepoint T0, all pairs from timepoint T1 and T2 were included for the analysis of paired kernels during inference.

**Table 1:** Reconstruction kernel pairs in the NLST across all timepoints.

Manufacturer	Kernel pair	Timepoint T0	Timepoint T1	Timepoint T2
**Siemens**	**(B30f, B50f)**	2786	3710	3514
	**(B30f, B80f)**	458	89	39
**GE**	**(STANDARD, BONE)**	3967	3454	3472
	**(STANDARD, LUNG)**	1021	871	678
**Philips**	**(C, D)**	1128	662	609
	**(B, D)**	168	113	135

Six different reconstruction kernel pairs are obtained across three different timepoints in the NLST post quality control. Data from timepoint T0 is considered for training the harmonization model while all pairs from timepoint T1 and T2 are used during inference.

**Table 2:** Kernel pairs at timepoint T0, split at the subject level for training the harmonization model.

Manufacturer	Kernel pair at baseline T0	Training data pairs	Validation data pairs	Testing data pairs
**Siemens**	**(B30f, B50f)**	1950	100	736
	**(B30f, B80f)**	321	50	87
**GE**	**(STANDARD, BONE)**	2777	100	1090
	**(STANDARD, LUNG)**	715	100	206
**Philips**	**(C, D)**	791	100	237
	**(B, D)**	118	25	25

All the kernel pairs at baseline T0 were split into training, validation and testing at the subject level. The counts represent the total number of hard and soft kernel image pairs. Validation data were used to pick the optimal checkpoint while testing data was used during inference of paired kernel harmonization.

#### 2.3.3 Kernel harmonization using anatomy guided multipath cycleGAN.

Recent advances in multipath cycleGAN architectures enable multi-domain kernel harmonization within a unified framework where a shared latent representation captures anatomy and style [21]. Unlike conventional cycleGAN models that require independent networks for each kernel combination, a multipath cycleGAN architecture scales efficiently in a multi-vendor lung screening dataset like the NLST, consisting of nine reconstruction kernels from three different vendors.

We adapted an anatomically guided multipath cycleGAN [23] for multi-vendor kernel harmonization across paired and unpaired reconstruction kernels in the NLST. The model consists of domain specific encoders and decoders connected through a shared latent space, enabling translation between any source and target kernel. We incorporate anatomical guidance using segmentation masks derived from TotalSegmentator [26] (version 2.8.0) that capture all available regions of interest in the image. This entails a tissue statistic loss that penalizes differences in the mean Hounsfield units between the source image and harmonized image to prevent inversion of tissue intensity in any given path.

Previously, the anatomy guided multipath cycleGAN was trained on four reconstruction kernels from the NLST [23]. We retrained this model to include all nine representative kernels by adopting a two-stage training approach to facilitate multi-vendor kernel harmonization. In stage one, Siemens and GE kernels were trained jointly using domain specific encoder-decoders and discriminators. In stage two, Philips kernels were introduced by freezing stage one weights and training additional networks to prevent catastrophic forgetting, allowing for domain adaptation (S2 Fig).

We implemented the model in PyTorch (version 2.5.1) with CUDA 12.4. We train our model on axial slices of size 512 × 512 pixels that are normalized to [−1,1]. Stage one was trained for 200 epochs on an NVIDIA RTX 6000 Ada Generation GPU (48GB RAM) using a batch size of one, Adam optimizer [32] and a learning rate of 2 × 10^−4^ that remains constant for 100 epochs before linearly decaying by a rate of 1.98 × 10^−6^. We trained stage one using 5000 randomly sampled slices from each domain, owing to the large dataset across multiple domains. In stage two, training across all datasets is computationally expensive owing to the number of image slices across all domains in addition to the encoder-decoder and discriminator architectures. Therefore, stage two was trained using the same configuration with 2000 randomly sampled slices from each domain. Each path in the network was governed by a least squares GAN (LSGAN) [33] loss function, a cycle consistency loss (mean absolute error (MAE) weighted by a factor of 10) and the tissue statistic loss (mean squared error (MSE) weighted by a factor of 1). In each path, forward translation maps the source kernel to the target kernel while backward translation maps the target kernel to the source kernel.

During inference, paired kernels were harmonized to the subject matched soft kernel within each vendor. At each cross sectional timepoint, all kernels were harmonized to the style of Siemens B30f (soft kernel) and Siemens B50f (hard kernel), yielding two standardized outputs per scan. All implementation details about model training, evaluation and analysis are provided at https://github.com/MASILab/Harmonized_NLST_dataset.

#### 2.3.4 Semantic field of view extension.

Thoracic CT scans in the NLST enable opportunistic body composition assessment of skeletal muscle and SAT. However, variable truncation patterns limit the FOV leading to underestimation of body composition measurements. Therefore, we perform semantic field of view extension using the algorithm developed by Xu et al.[20]. The algorithm follows a two-stage framework: stage one focuses on predicting a bounding box that estimates the extent of the complete body while stage two imputes the truncated anatomical regions using the truncated input image and the corresponding FOV mask, treating semantic FOV extension as an out painting task. We apply FOV extension slice-wise to all the images post kernel harmonization and stack all slices to obtain the semantically extended volume.

#### 2.3.5 Downstream analysis.

Using TotalSegmentator (version 2.8.0), we derive segmentation labels on the semantically extended images. All default labels were obtained, followed by skeletal muscle and SAT segmentations using the tissue types model. We compute percent emphysema using the lung segmentation masks from TotalSegmentator. Emphysema can be characterized by regions in the lung that have a radio density range from −900 to −1024 HU [34]. Therefore, emphysema was quantified as the percentage of voxels with attenuation less than −950 HU. Skeletal muscle and SAT volumes were computed by multiplying the voxel counts with the voxel volume obtained from the image header.

### 2.4 Evaluation and statistical analysis

We evaluate the performance of our standardization pipeline across three different settings: a) paired reconstruction kernels at each timepoint b) cross sectional cohorts across all kernels at each timepoint and c) longitudinal scans acquired from the same subject over time. For paired reconstruction kernels, we assess agreement in percent emphysema, skeletal muscle and body composition assessment before and after harmonization. We report the root mean square error (RMSE) that quantifies agreement along with the 95% confidence intervals while Bland Altman [35] style plots are used to visualize the agreement between measures. Differences in measurements were computed as hard kernel minus soft kernel for all pairs. 95% confidence intervals were computed through bootstrapping with replacement using 1000 resamples. Body composition assessment was performed post FOV extension. At the image level, we evaluate image similarity metrics using RMSE, peak signal to noise ratio (PSNR) and structural similarity index (SSIM) for intensity windows corresponding to lung ([−1000,0] HU), muscle ([−29, 150] HU) and SAT ([−190, −30]). Two-sample, two-sided, Wilcoxon signed ranked tests were used to determine significant changes in distributions of measures before and after harmonization. To account for multiple comparisons, p-values were adjusted using the Benjamini-Hochberg false discovery rate correction (*p* < 0.05).

For cross sectional analysis, at each timepoint, we matched cohorts on age, sex and smoking status using exact matching to reduce demographic confounding. To ensure balanced comparison across kernels, we used the kernel with the lowest sample size for matching at a given timepoint. Using the matched cohorts, we quantify the effect size between the emphysema distributions of the reference kernel and each source kernel before and after harmonization using Cohen’s d [36]. Effect sizes are small when *d* = 0.2, medium when *d* = 0.5 and large when *d* ≥ 0.8. A positive *d* indicates that group one had a higher mean compared to group two while a negative *d* indicates the opposite. The reference kernels for harmonization were Siemens B50f and B30f. For body composition analysis, we use the data from all kernels across all timepoints, computing the SAT ratio before and after FOV extension as the ratio of SAT volume to the combined skeletal muscle and SAT volume. The agreement between truncated and FOV extended measurements were evaluated using Bland-Altman analysis of SAT ratios to quantify the systematic increases associated with the recovery of missing tissue. Furthermore, we evaluate body composition assessment by examining the association between SAT and skeletal muscle indices (cm^3^/m^2^) and their corresponding anthropometric measures (fat mass for SAT and fat free mass for skeletal muscle) following Xu et al [20]. We reported the spearman rank correlation and 95% confidence intervals computed through bootstrapping with 1000 resamples (with replacement). At timepoints T1 and T2, we used the approximate anthropometric measures obtained at baseline for correlation analysis.

At the longitudinal level, we are interested in quantifying the stability of biomarkers across time for each subject post harmonization. We model longitudinal trajectories using a linear mixed effects model of the form:


$$\begin{array}{c} y_{ij}\;\sim \;\beta_{\text{0}}{+\;\beta }_{\text{1}}{Timepoint}_{ij}\;{+\;\beta }_{\text{2}}{{Age}_{ij}+\beta }_{\text{3}}{Sex}_{i\;}{+\;\beta }_{\text{4}}{Smoking}_{ij}{\;+\;\beta }_{\text{5}}{Kernel}_{ij}\;{+\;r}_{i\;}{+\;\varepsilon }_{ij} \end{array}$$ (1)

where $y_{ij}$ is the biomarker of interest (emphysema, SAT index) for subject i at scan j, $\beta_{\text{0}}$ is the population average at baseline timepoint T0, ${Timepoint}_{ij}$ is the longitudinal timepoint (T1, T2) for subject i at scan j, ${Age}_{ij}$ corresponds to age at baseline for subject i at scan j, ${Sex}_{i}$ is the male/female sex of subject i where male is encoded as 0 and female is encoded as 1, ${Smoking}_{ij}$ is the smoking status for subject i at scan j and ${Kernel}_{ij}$ is the reconstruction kernel used for scan j of subject i. We model subject-specific variability as a random $r_{i}\;\sim N\left(\text{0},\;\tau^{\text{2}}\right)$, where $\tau^{\text{2}}$ is the estimated between-subject variance. Finally, $\varepsilon_{ij}\;\sim \;N\left(\text{0},\;\sigma^{\text{2}}\right)$ denotes the residual error term for scan j of subject i, where $\sigma^{\text{2}}$ is the residual population variance. We monitor longitudinal stability using the intra-class correlation coefficient (ICC), computed as the ratio:


$$\begin{array}{c} \frac{\tau^{\text{2}}}{\tau^{\text{2}}+\sigma^{\text{2}}} \end{array}$$ (2)

## 3 Results

Post harmonization and FOV extension, we manually inspected all images through visual assessment of the triplanar views for each scan to ensure consistent anatomy for downstream analysis. We excluded a total 999 images harmonized to Siemens B30f and 676 images harmonized to Siemens B50f that failed the quality assurance.

### 3.1 Paired reconstruction kernels

The impact of kernel harmonization at the image level was assessed using image similarity metrics for lung, muscle and SAT respectively. We present the voxel-wise RMSE, PSNR, and SSIM for all three regions of interest across timepoints T0, T1 and T2 in Table 3 and supplementary tables **ST3** and **ST**4 in S1 File, expressed as the mean and standard deviation**.** All results are summarized as the percentage reduction in RMSE and the percentage increases in PSNR and SSIM. Across all timepoints, most kernel pairs showed large improvements in agreement for all metrics post harmonization, with smaller improvements observed for (C,D) and (B,D) in certain anatomical regions. For the lung region, the (B30f, B80f) pair consistently showed the largest improvement, achieving the largest reduction in RMSE (T0:70.8%, T1:74.4%, T2:73.6%), largest increase in PSNR (T0:50.9%, T1:60.1%, 58.8%) and SSIM (T0:77.4%, T1:82.4%, T2:82.4%). A similar trend was observed for skeletal muscle where the (B30f, B80f) pair yielded the largest RMSE reduction (T0:66.8%, T1:66.7%, T2:66.6%) and increases in PSNR (T0:76.8%, T1:81.2%, T2:80.4%) and SSIM (T0:54.4%, T1:58.2%, T2:58.2%). For SAT, (B30f, B80f) once again achieved the best performance for RMSE (T0:71%,T1:69.7%,T2:69%), PSNR (T0:87.3%, T1:90.8%, T2:88.8%) and SSIM (T0:52.5%, T1:50.8%,T2:46.8%).

**Table 3:** Image similarity metrics obtained for paired kernels at timepoint T0.

Region of interest	Kernel pairs	RMSE		PSNR		SSIM	
		Before	After	Before	After	Before	After
**Lung**	**(B30f, B50f)**	43.31 (10.06)	27.66 (4.21)	27.45 (1.73)	31.25 (1.22)	0.73 (0.05)	0.94 (0.02)
	**(B30f, B80f)**	94.41 (22.02)	27.55 (3.17)	20.70 (1.80)	31.25 (0.91)	0.53 (0.04)	0.94 (0.02)
	**(STANDARD,** **BONE)**	56.23 (17.12)	37.09 (5.57)	25.36 (2.47)	28.71 (1.26)	0.68 (0.06)	0.88 (0.05)
	**(STANDARD,** **LUNG)**	75.22 (29.32)	47.15 (7.19)	23.00 (2.89)	26.62 (1.23)	0.63 (0.08)	0.87 (0.06)
	**(C, D)**	37.82 (9.58)	36.98 (6.78)	28.69 (1.97)	28.77 (1.47)	0.83 (0.09)	0.87 (0.05)
	**(B, D)**	39.14 (11.60)	37.20 (6.25)	28.48 (2.33)	28.69 (1.25)	0.83 (0.10)	0.87 (0.05)
**Skeletal muscle**	**(B30f, B50f)**	26.39 (4.43)	14.47 (2.73)	16.74 (1.40)	21.98 (1.47)	0.71 (0.06)	0.87 (0.03)
	**(B30f, B80f)**	42.56 (5.18)	14.14 (2.53)	12.54 (1.02)	22.17 (1.43)	0.57 (0.07)	0.88 (0.03)
	**(STANDARD,** **BONE)**	32.19 (6.00)	18.85 (4.61)	15.05 (1.61)	19.79 (2.03)	0.67 (0.07)	0.82 (0.04)
	**(STANDARD,** **LUNG)**	36.29 (6.60)	21.00 (5.82)	14.00 (1.53)	18.90 (2.15)	0.62 (0.07)	0.79 (0.06)
	**(C, D)**	24.25 (4.10)	23.32 (5.34)	17.48 (1.40)	17.91 (1.97)	0.73 (0.06)	0.77 (0.06)
	**(B, D)**	24.83 (5.37)	20.28 (4.80)	17.35 (1.82)	19.15 (2.01)	0.72 (0.07)	0.78 (0.05)
**Subcutaneous adipose tissue**	**(B30f, B50f)**	23.37 (5.28)	12.39 (2.79)	16.93 (1.98)	22.40 (1.69)	0.74 (0.04)	0.92 (0.03)
	**(B30f, B80f)**	38.49 (5.84)	11.17 (3.06)	12.48 (1.34)	23.38 (1.99)	0.61 (0.04)	0.93 (0.03)
	**(STANDARD,** **BONE)**	29.39 (6.65)	16.82 (4.60)	14.95 (2.03)	19.85 (2.17)	0.72 (0.05)	0.85 (0.05)
	**(STANDARD,** **LUNG)**	31.63 (7.88)	18.71 (5.24)	14.36 (2.21)	18.92 (2.08)	0.69 (0.05)	0.84 (0.07)
	**(C, D)**	19.12 (5.43)	18.02 (4.79)	18.78 (2.37)	19.25 (2.20)	0.82 (0.06)	0.84 (0.05)
	**(B, D)**	19.53 (6.37)	16.55 (3.94)	18.69 (2.65)	19.94 (1.99)	0.80 (0.08)	0.84 (0.07)

Image similarity metrics computed for lung window ([−1000,0] Hounsfield units), subcutaneous adipose tissue window ([−190,-30] Hounsfield units) and skeletal muscle window ([−29,150] Hounsfield units) for reconstruction kernels in the NLST at timepoint T0. All metrics are expressed as the mean with standard deviation in parentheses. RMSE values decrease, PSNR and SSIM values increase post harmonization. Wilcoxon signed ranked test is used to test for significance before and after harmonization. The Benjamini-Hochberg procedure was applied to adjust for multiple comparisons and control the false discovery rate. All values except the RMSE and PSNR computed for the lung for the pairs (B,D) and (C,D) were statistically significant (*p* < 0.05).

To analyse the complexity of our proposed GAN approach, we briefly compare its performance against a simple histogram matching on image similarity metrics obtained on the lung, muscle and SAT windows at timepoint T0. All results are reported as the mean and standard deviation in supplementary tables **ST5**, **ST6** and **ST7** in S1 File. Across all three regions, our proposed model outperformed the baseline across all metrics for the (B30f, B50f), (B30f, B80f), (STANDARD, BONE) and (STANDARD, LUNG) pairs. However, on the (B, D) and (C, D) pairs, the baseline achieved a larger reduction in RMSE across the lung and skeletal muscle while our approach was slightly better only on the (B,D) pair for SAT. PSNR followed a similar pattern: the baseline generally achieved larger improvements in the lung and skeletal muscle regions, whereas our approach performed better on the (B, D) pair for SAT. For SSIM, our approach outperformed the baseline in the lung and SAT regions while the measures for skeletal muscle were comparable.

We evaluated the impact of harmonization on downstream quantitative measures, including percent emphysema, skeletal muscle volume and SAT volume across all timepoints. Results are reported as the RMSE and 95% confidence intervals in Table 4 (timepoint T0) and supplementary tables **ST8** in S1 File (timepoint T1) and **ST9** in S1 File (timepoint T2). Before harmonization, hard kernels overestimate percent emphysema measures as compared to soft kernels due to the amplification of voxel noise [37]. Harmonization reduces the differences in percent emphysema across all kernel pairs and timepoints, leading to improved consistency in measures as seen from the violin plots (Fig 4). This is further supported by the Bland Altman analysis for the (STANDARD, BONE) pair that has the largest sample size across all pairs and timepoints (Fig 5). Among all the pairs, (STANDARD, LUNG) demonstrated the greatest reduction in percent emphysema differences (T0: 19.45% [18.77%, 20.10%] to 1.21% [0.93%, 1.61%], T1: 19.53% [19.23%, 19.82%] to 1.45% [1.29%, 1.64%], and T2: 19.83% [19.51%, 20.16%] to 1.52% [1.36%, 1.76%]).

**Table 4:** Emphysema and body composition measures for paired kernels at timepoint T0.

Kernel pairs	Emphysema (%)		Skeletal muscle (cm^3^)		Subcutaneous adipose tissue (cm^3^)	
	Before	After	Before	After	Before	After
**(B30f,** **B50f)**	11.42 [11.25, 11.60]	1.60 [1.49, 1.82]	135.46 [107.31, 219.53]	46.59 [43.05, 54.37]	307.61 [242.10, 463.52]	58.45 [51.10, 77.92]
**(B30f,** **B80f)**	28.08 [27, 28.92]	2.98 [2.66,3.37]	144.99 [107.63, 191.41]	96.35 [80.36, 120.52]	582.67 [415.80, 811.05]	97.85 [82.02, 114.84]
**(STANDARD, BONE)**	9.43 [9.24, 9.61]	1.09 [1.02, 1.17]	197.80 [187.70, 215.91]	54.74 [49.30, 65.85]	458.13 [426.66, 499.61]	99.91 [86.48, 125.66]
**(STANDARD, LUNG)**	19.45 [18.77, 20.10]	1.21 [0.93, 1.61]	248.47 [188.93, 345.36]	56.02 [43.29, 76.70]	798.93 [650.67, 1040.69]	73.33 [63.38, 87.80]
**(C,D)**	15.77 [15.12, 16.46]	2.84 [2.59, 3.15]	126.70 [114.90, 142.75]	119.57 [108.73, 137.23]	294.76 [268.77, 328.98]	129.35 [111.50, 163.57]
**(B,D)**	14.05 [12.00, 16.34]	2.07 [0.75, 4.66]	157.81 [102.39, 245.06]	134.43 [107.45, 167.09]	307.48 [238.58, 398.00]	144.50 [114.21, 184.16]

Percent emphysema, skeletal muscle volume and subcutaneous adipose tissue volumes computed for reconstruction kernel pairs at timepoint T0. The measures are expressed as the root mean square error (RMSE) with 95% confidence intervals in square brackets. RMSE values decrease post harmonization for all pairs. Statistical significance for all measurements before and after harmonization were determined by a Wilcoxon signed rank test with bootstrapping (*p* < 0.05). The Benjamini-Hochberg procedure was applied to adjust for multiple comparisons and control the false discovery rate. For emphysema and subcutaneous adipose tissue, all pairs were significant. For skeletal muscle, all pairs except (B30f, B80f), (C, D) and (B, D) were significant.

**Fig 4:**
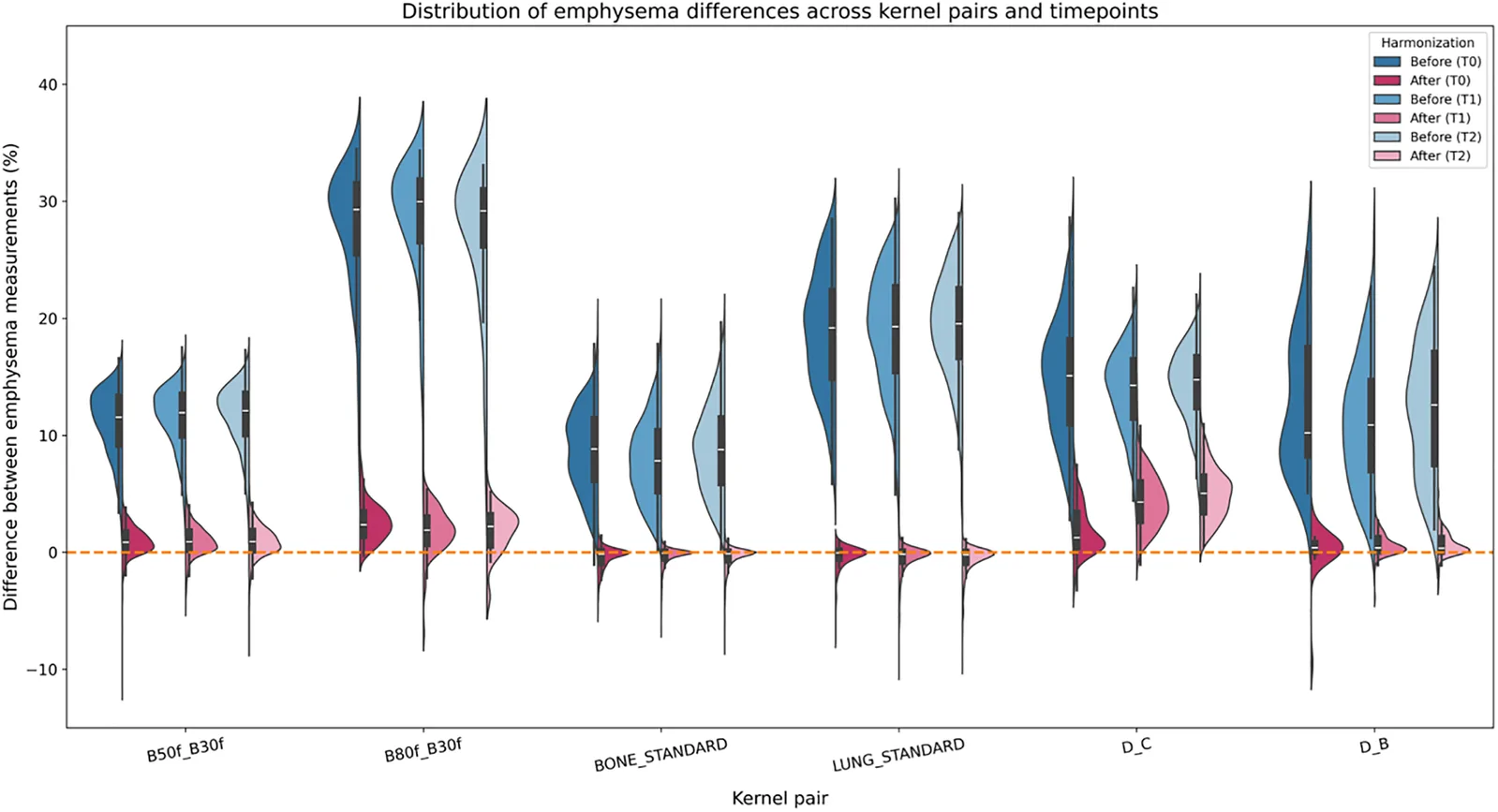
Percent emphysema distributions for paired kernels across all timepoints. Paired reconstruction kernels across different timepoints in the NLST exhibit variability in percent emphysema measurements. Differences in percent emphysema before harmonization demonstrate that hard kernels overestimate emphysema as compared to soft kernels, reflected by positive medians and wide distributions. Harmonization to the corresponding soft kernel yields narrower distributions with decreased variability, indicating improved consistency in percent emphysema measurements across all kernel pairs and timepoints.

**Fig 5:**
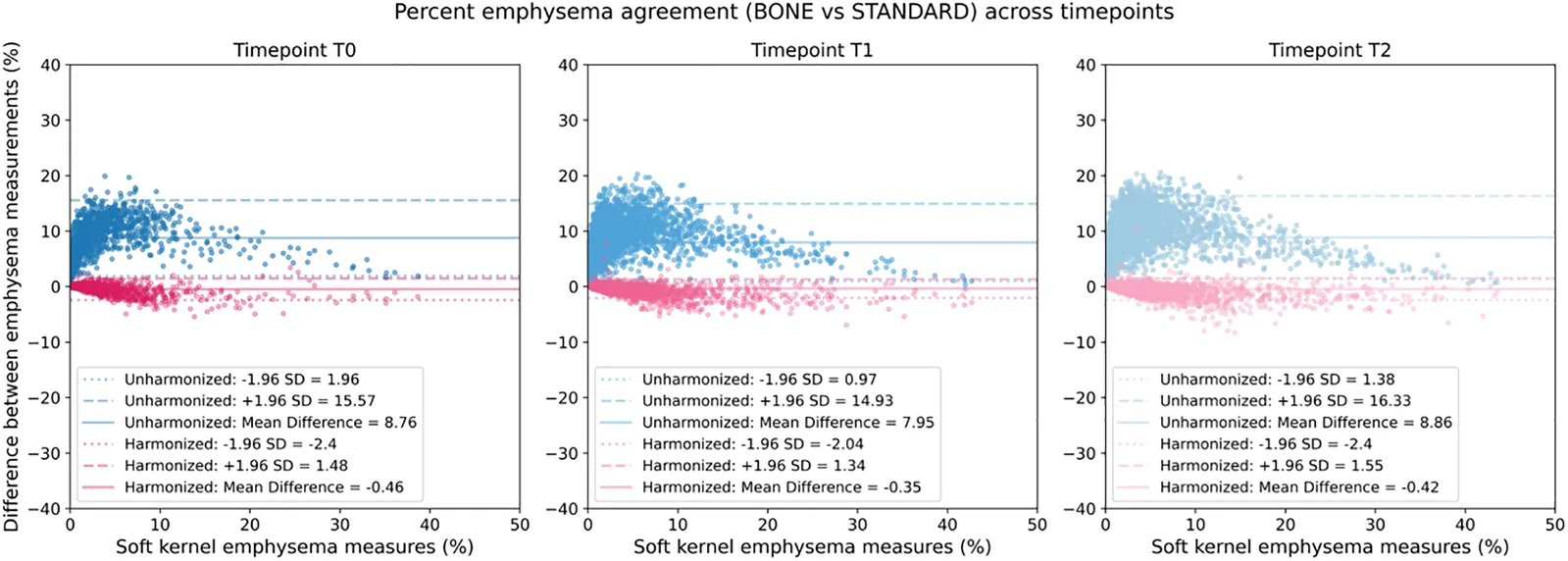
Bland Altman style plots for percent emphysema across all timepoints. We use Bland Altman style plots for the (BONE, STANDARD) pair across all timepoints to represent the percent emphysema agreement between paired hard (BONE) and soft (STANDARD) reconstruction kernels before and after harmonization. Solid lines denote the mean difference (bias) and dashed lines represent the limits of agreement (±1.96 standard deviation). Before harmonization, the BONE kernel overestimates emphysema, as indicated by the positive bias and wider limits of agreement. Harmonization to the STANDARD kernel minimizes differences in measures, as seen from a lower bias and narrower limits of agreement.

Differences in skeletal muscle volume exhibit positive and negative deviations before harmonization across all kernel pairs as seen in the violin plot distributions in Fig 6. Harmonization reduced variability across most pairs and timepoints, resulting in tighter distributions and improved agreement, as illustrated for the (STANDARD, BONE) pair (Fig 7). However, (B,D) and (C,D) continued to exhibit residual bias at timepoint T0. The largest reductions in skeletal muscle volume were observed for the (STANDARD, LUNG) pair at timepoints T0 (248.47 cm^3^ [188.93 cm^3^, 345.36 cm^3^] to 56.02 cm^3^ [43.29 cm^3^, 76.7 cm^3^]) and T1(227.52 cm^3^ [200.15 cm^3^, 276.63 cm^3^] to 56.6 cm^3^ [50.45 cm^3^, 69.65 cm^3^]) while the (B30f, B80f) achieved the largest reduction at timepoint T2 (361.01 cm^3^ [137.05 cm^3^, 734.96 cm^3^] to 58.87 cm^3^ [44.51 cm^3^, 81.2 cm^3^]). For SAT volume, hard kernels consistently underestimate volumes as observed from the negative medians and wider distributions observed in Fig 8 and the negative bias for the (STANDARD, BONE) pair in Fig 9. Harmonization reduces the differences and improved consistency across all pairs. The (STANDARD, LUNG) pair achieved the largest reduction in at timepoint T0 (798.93 cm^3^ [650.67 cm^3^, 1040.69 cm^3^] to 73.33 cm^3^ [63.38 cm^3^, 87.8 cm^3^]) and timepoint T1 (798.13 cm^3^ [718.13 cm^3^, 923.55 cm^3^] to 78.14 cm^3^ [68.57 cm^3^, 98.26 cm^3^]), while (B30f,B80f) exhibited the largest reduction in SAT volume at timepoint T2 (952.52 cm^3^ [491.22 cm^3^, 1598.13 cm^3^] to 92.36 cm^3^ [63.25 cm^3^, 147.43 cm^3^]).

**Fig 6:**
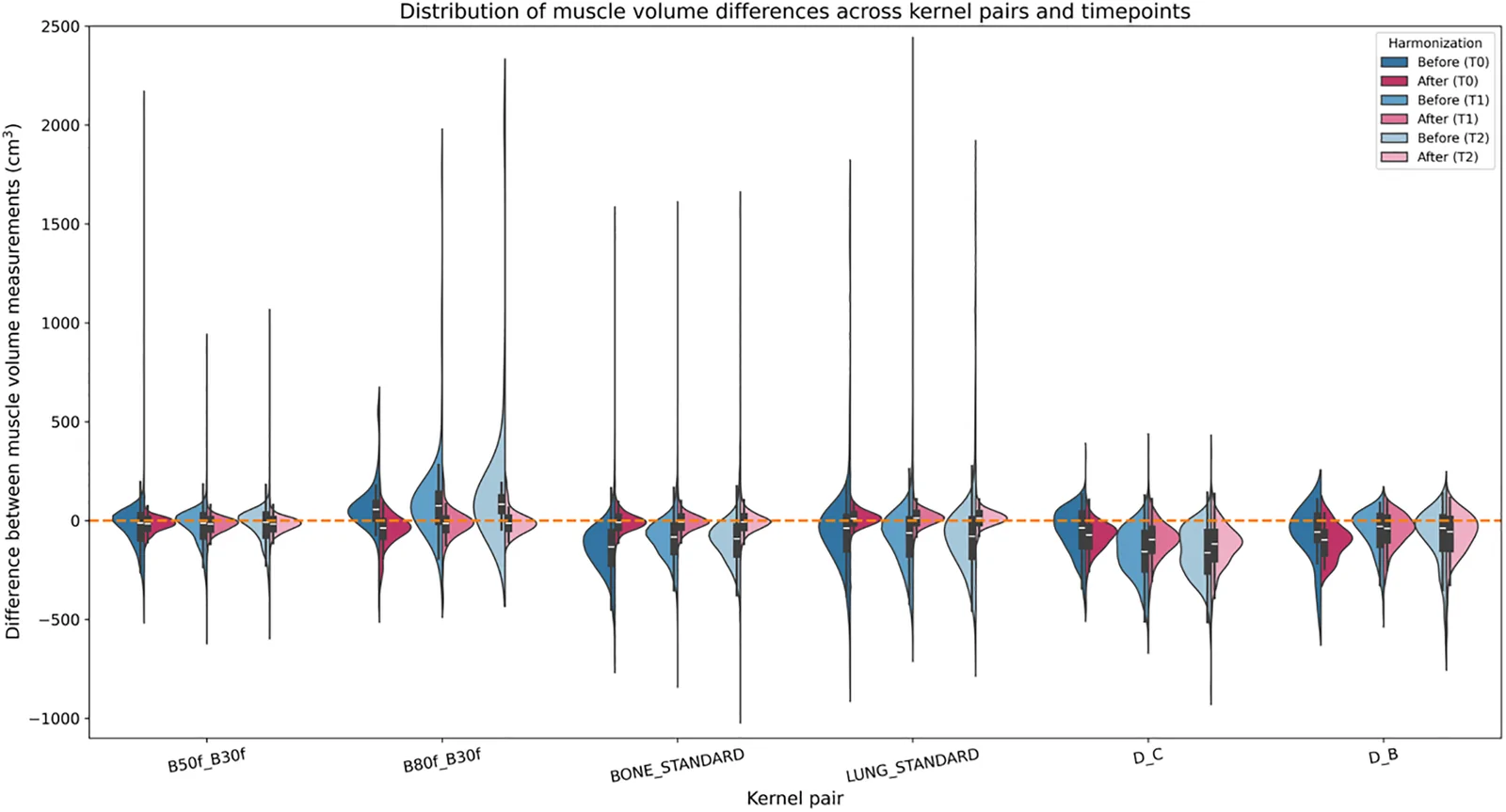
Skeletal muscle volume distributions for paired kernels across all timepoints. Differences in skeletal muscle volumes post FOV extension across all kernel pairs and timepoints in the NLST do not exhibit consistent directional bias, with positive and negative deviations observed before harmonization. Variability in measures are higher before harmonization, reflected by wider distributions with extreme tails. Harmonization minimizes variability, resulting in narrower distributions and improved consistency in muscle volume measurements across kernel pairs and timepoints, although residual differences persist in some pairs.

**Fig 7:**
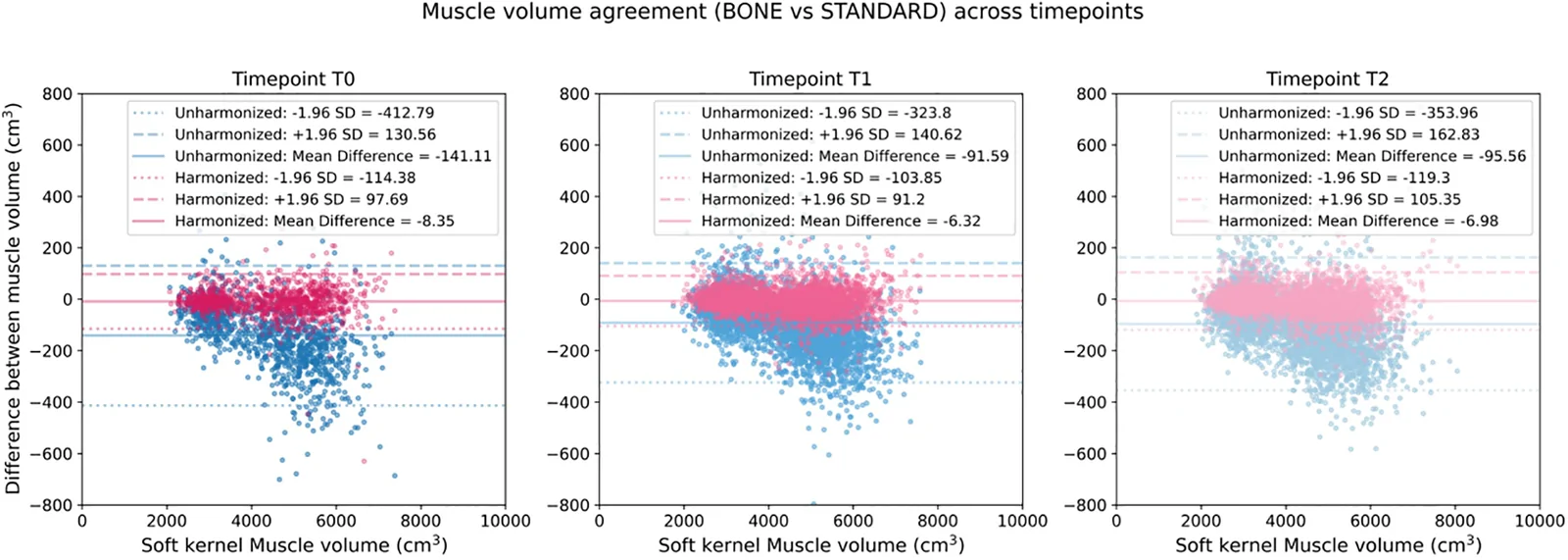
Bland Altman style plots for skeletal muscle volume across all timepoints. Bland-Altman style plots are used to represent agreement between hard and soft kernels for the (BONE, STANDARD) pair on skeletal muscle volume. We present mean difference (bias) and limits of agreement before and after harmonization. Prior to harmonization, the BONE kernel underestimates skeletal muscle volume compared to the STANDARD kernel, resulting in a negative bias and wider limits of agreement. Harmonization to the STANDARD kernel minimizes bias in measurements, suggesting that volumes are more consistent across all timepoints.

**Fig 8:**
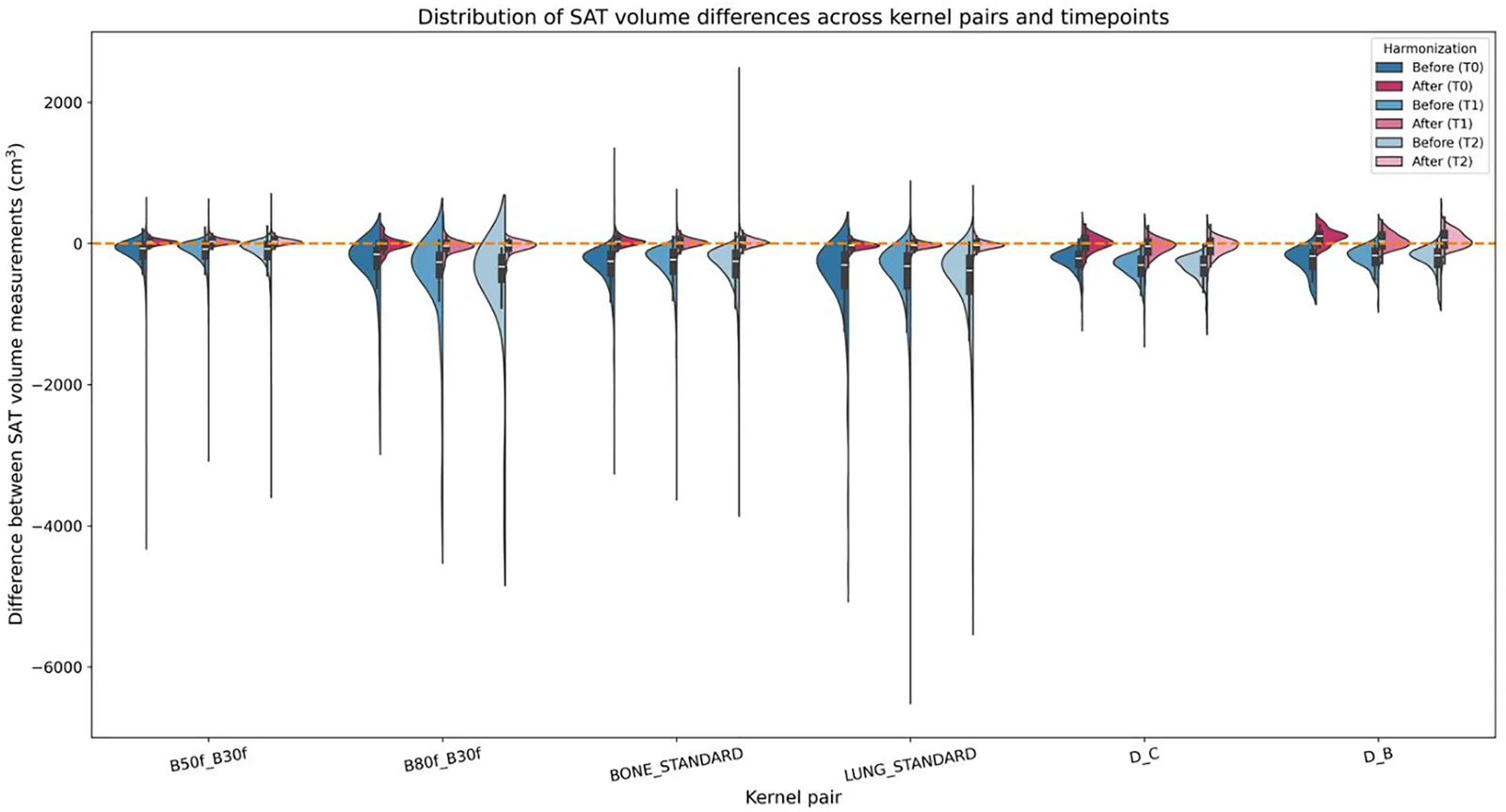
Subcutaneous adipose tissue volumes for paired kernels across all timepoints. Distribution of differences in subcutaneous adipose tissue (SAT) volumes across all kernel pairs and timepoints in the NLST exhibit predominantly negative medians and wider distributions with extreme tails. This suggests that hard kernels underestimate volumes compared to soft kernels. Harmonization minimizes differences in volumes, shifting the distributions closer to zero with a narrower spread, indicating that measurements are more consistent across all kernel pairs and timepoints.

**Fig 9:**
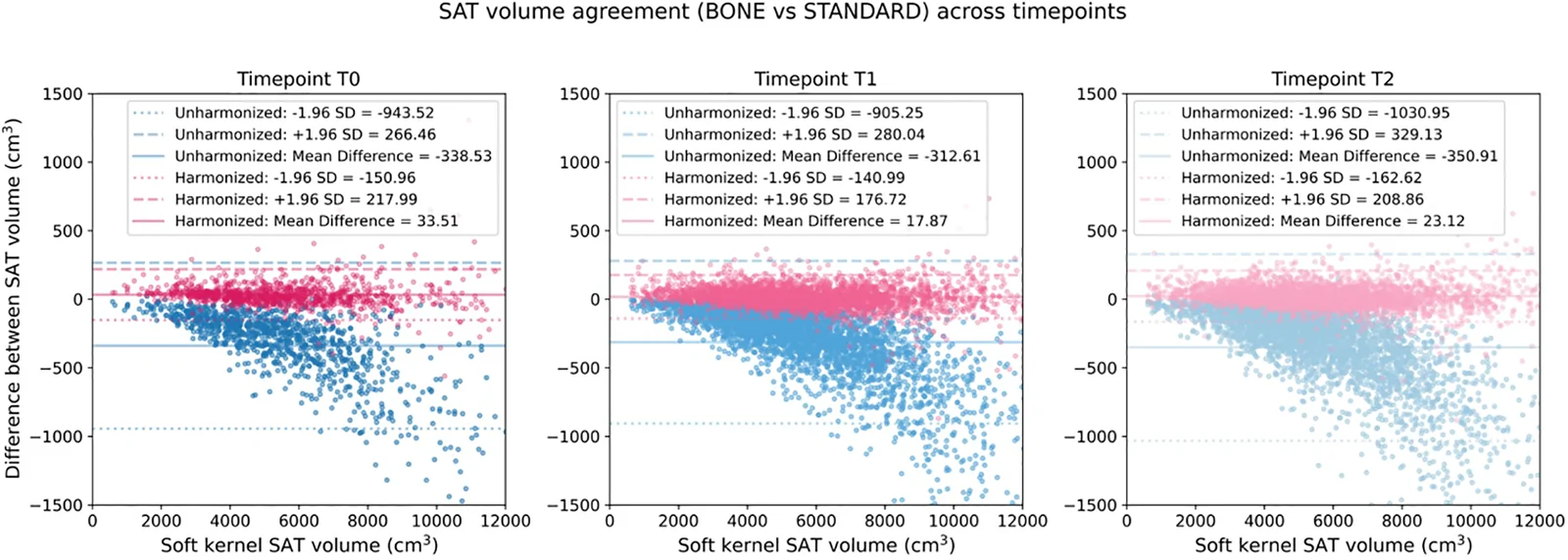
Bland Altman style plots for subcutaneous adipose tissue volume across all timepoints. We use Bland-Altman plots to assess agreement between SAT volumes derived from hard (BONE) and soft (STANDARD) kernels for the (BONE,STANDARD) across all timepoints. Before harmonization, the BONE kernel underestimates SAT volumes relative to the STANDARD kernel, indicated by a negative mean difference and wider limits of agreement. Harmonization to the STANDARD kernel minimizes the bias and leads to narrower limits of agreement, showcasing consistent measures across all timepoints.

### 3.2 Cross sectional analysis

At each timepoint, we assessed the impact of harmonization by harmonizing images from all kernels to Siemens B30f (soft kernel) and Siemens B50f (hard kernel), evaluating percent emphysema on cohorts matched by age, sex and smoking status across all kernels. Before harmonization, hard kernels overestimate percent emphysema as compared to soft kernels across all timepoints (Fig 10). Harmonization to the style of the reference B30f and B50f kernels shifts the distributions closer to the reference, aligning the medians across kernels. We quantify the magnitude of kernel dependent differences in emphysema distributions before and after harmonization using Cohen’s *d* presented in Table 5 and supplementary tables **ST10** and **ST11** in S1 File. When harmonized to the Siemens B30f soft kernel, hard kernels demonstrated the largest reduction in effect sizes which included Siemens B50f (T0: −1.73 to −0.27, T1: −1.51 to −0.10, T2: −1.85 to −0.31), GE LUNG (T0: −1.96 to −0.21, T1: −1.61 to 0.01, T2: −1.73 to −0.06) and Siemens B80f (T0: −4.10 to −0.35, T1: −3.92 to −0.21, T2: −4.03 to −0.53). Among the soft kernels, Philips C showed consistent improvement across all timepoints (T0: 0.60 to 0.19, T1: 0.93 to 0.43, T2: 0.61 to 0.19). For kernels harmonized to the Siemens B50f hard kernel, both soft and hard kernels showed considerable improvements. Siemens B30f (T0: 1.73 to 0.30, T1: 1.51 to 0.24, T2: 1.85 to 0.48), GE STANDARD (T0: 2.60 to 0.14, T1: 2.80 to 0.25, T2: 3.00 to 0.42) and Philips C (T0: 2.58 to 0.17, T1: 3.08 to 0.25, T2: 3.01 to 0.39) showed consistent improvement across all timepoints among the soft kernels while Siemens B80f (T0: −2.37 to −0.251, T1: −2.48 to −0.30, T2: −2.42 to −0.32) and GE BONE (T0: 1.07 to 0.24, T1: 1.38 to 0.38, T2: 1.01 to 0.01) demonstrated largest improvements among the hard kernels.

**Table 5:** Cohen’s *d* effect sizes computed on matched samples at timepoint T0.

Reference kernel	Reconstruction kernel	Cohen’s *d* before harmonization	Cohen’s *d* after harmonization
**Siemens B30f**	**Siemens B50f**	−1.73	−0.27
	**Siemens B80f**	−4.10	−0.36
	**GE BONE**	−0.68	−0.25
	**GE STANDARD**	0.62	−0.43
	**GE LUNG**	−1.96	−0.21
	**Philips B**	0.58	0.13
	**Philips C**	0.60	0.19
	**Philips D**	−1.45	−0.54
**Siemens B50f**	**Siemens B30f**	1.73	0.30
	**Siemens B80f**	−2.37	−0.25
	**GE BONE**	1.07	0.24
	**GE STANDARD**	2.60	0.14
	**GE LUNG**	−0.27	0.05
	**Philips B**	2.48	0.33
	**Philips C**	2.58	0.17
	**Philips D**	0.09	0.10

We present Cohen’s *d* effect sizes computed between the emphysema distributions of the reference kernel and source kernel before and after harmonization for matched samples at timepoint T0. Cohen’s *d* quantifies the magnitude of difference between the emphysema scores, highlighting kernel bias. Effect sizes are small when *d* = 0.2, medium when *d* = 0.5 and large when *d* ≥ 0.8. A positive *d* indicates that group one had a higher mean compared to group two while a negative *d* indicates the opposite. Harmonization to the reference reduces bias in emphysema measures as evidenced by a shift from large effect sizes to small or medium effect sizes across different kernels.

**Fig 10:**
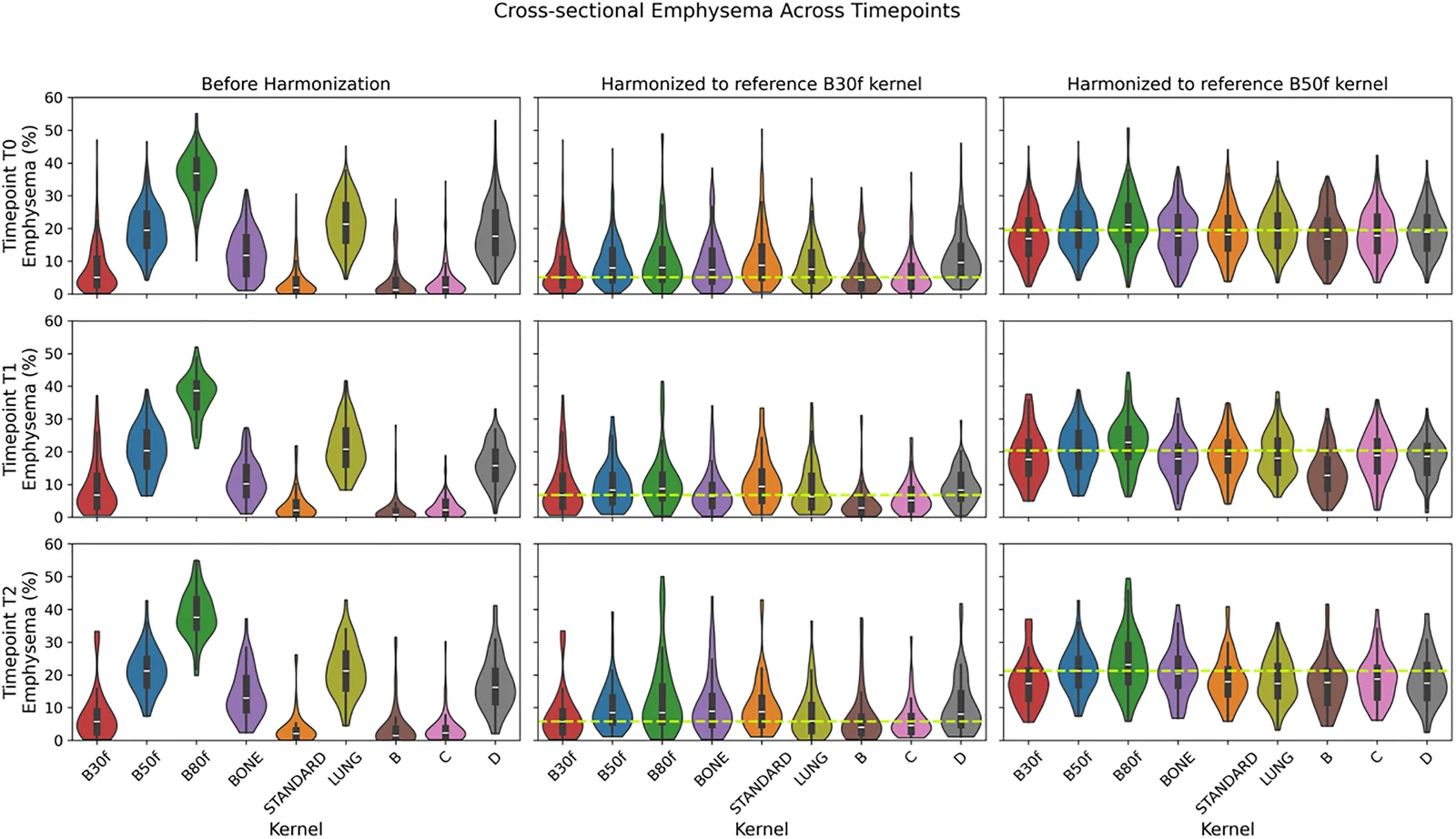
Percent emphysema distributions for matched samples before and after harmonization. We present the cross-sectional emphysema distributions across all timepoints in the NLST on subjects matched by age, sex and smoking status. Before harmonization, emphysema distributions vary substantially across kernels, with hard kernels yielding higher emphysema scores compared to soft kernels. Harmonization to the reference Siemens B30f kernel aligns distributions across all kernels, minimizing variability and bringing the medians closer to the reference. Similarly, harmonization to the reference Siemens B50f kernel results in consistent distributions across all kernels with medians converging to the reference distribution.

We evaluated body composition on the semantically extended images post harmonization. Specifically, we computed SAT ratios before and after FOV extension to assess the recovery of missing tissue due to truncation. Bland Altman analysis of SAT ratios demonstrated a positive mean difference across all vendors and timepoints (Figs 11 and 12). The magnitude of the mean difference varied across vendors, with GE demonstrating the largest mean difference across timepoints, followed by Siemens and Philips. To further validate body composition, we examined the correlation of body composition measures with anthropometric measures for all the kernels across different vendors. Statistical significance between correlations was determined using the method developed by Hitner et al [38]. We present the correlations for muscle index and SAT index with fat free mass (FFM) and fat mass (FM) in Table 6 and supplementary tables **ST12** and **ST13** in S1 File for all images harmonized to Siemens B30f while correlations for images harmonized to B50f are presented in Table 7 and supplementary tables **ST14** and **ST15** in S1 File. Across all timepoints, FOV extension improved correlations for skeletal muscle and SAT with the GE LUNG, GE BONE and GE STANDARD images showing the largest improvement in correlations and the Philips and Siemens kernels showing minor improvements.

**Table 6:** Body composition correlations post harmonization to Siemens B30f and FOV extension at timepoint T0.

Kernel	SAT index (cm^3^/m^2^) vs FM index (kg/m^2^)			Muscle index (cm^3^/m^2^) vs FFM index (kg/m^2^)		
	Truncated	FOV extended	*p*-value	Truncated	FOV extended	*p*-value
**Siemens B30f**	0.838 [0.830,0.846]	0.856 [0.849,0.863]	<0.05	0.728 [0.716,0.739]	0.732 [0.720,0.743]	<0.05
**Siemens B50f**	0.823 [0.814,0.832]	0.856 [0.848,0.863]	<0.05	0.717 [0.703,0.729]	0.746 [0.734,0.757]	<0.05
**Siemens B80f**	0.859 [0.829,0.882]	0.880 [0.853,0.901]	<0.05	0.746 [0.703,0.783]	0.739 [0.693,0.779]	0.05
**GE BONE**	0.757 [0.747,0.767]	0.861 [0.853,0.867]	<0.05	0.689 [0.677,0.699]	0.729 [0.717,0.738]	<0.05
**GE STANDARD**	0.775 [0.767,0.782]	0.852 [0.846,0.857]	<0.05	0.668 [0.659,0.676]	0.727 [0.719,0.735]	<0.05
**GE LUNG**	0.744 [0.715,0.771]	0.852 [0.832,0.870]	<0.05	0.682 [0.650,0.712]	0.720 [0.691,0.746]	<0.05
**Philips B**	0.806 [0.745,0.854]	0.833 [0.781,0.873]	<0.05	0.728 [0.656,0.782]	0.744 [0.676,0.795]	<0.05
**Philips C**	0.818 [0.800,0.834]	0.842 [0.825,0.857]	<0.05	0.705 [0.680,0.727]	0.716 [0.691,0.738]	<0.05
**Philips D**	0.813 [0.787,0.833]	0.849 [0.828,0.866]	<0.05	0.679 [0.645,0.709]	0.706 [0.673,0.734]	<0.05

Comparison of body composition correlations between the body composition volume indices normalized by square of the height and the approximated anthropometric body composition indices before and after FOV extension at timepoint T0. FOV extension is performed on the images harmonized to the reference Siemens B30f kernel. The approximations were derived from the height and weight of the subject at baseline. We report the spearman rank correlations with 95% confidence intervals. Significance before and after FOV extension was evaluated using the method implemented by Hittner et al [38]. FM = Fat mass, FFM = Fat free mass, SAT = Subcutaneous adipose tissue.

**Table 7:** Body composition correlations post harmonization to Siemens B50f and FOV extension at timepoint T0.

Kernel	SAT index (cm^3^/m^2^) vs FM index (kg/m^2^)			Muscle index (cm^3^/m^2^) vs FFM index (kg/m^2^)		
	Truncated	FOV extended	*p*-value	Truncated	FOV extended	*p*-value
**Siemens B30f**	0.838 [0.830,0.847]	0.850 [0.843,0.858]	<0.05	0.728 [0.716,0.739]	0.734 [0.722,0.745]	<0.05
**Siemens B50f**	0.823 [0.813,0.832]	0.847 [0.838,0.855]	<0.05	0.717 [0.704,0.729]	0.730 [0.718,0.743]	<0.05
**Siemens B80f**	0.859 [0.829,0.882]	0.876 [0.848,0.897]	<0.05	0.746 [0.703,0.783]	0.736 [0.691,0.776]	<0.05
**GE BONE**	0.757 [0.746,0.768]	0.855 [0.846,0.862]	<0.05	0.689 [0.677,0.699]	0.736 [0.726,0.745]	<0.05
**GE STANDARD**	0.775 [0.767,0.782]	0.845 [0.839,0.851]	<0.05	0.668 [0.658,0.677]	0.729 [0.721,0.737]	<0.05
**GE LUNG**	0.746 [0.717,0.773]	0.846 [0.826,0.865]	<0.05	0.680 [0.650,0.711]	0.710 [0.680,0.738]	<0.05
**Philips B**	0.798 [0.736,0.849]	0.818 [0.763,0.861]	<0.05	0.731 [0.663,0.787]	0.705 [0.634,0.763]	<0.05
**Philips C**	0.817 [0.798,0.834]	0.834 [0.817,0.85]	<0.05	0.705 [0.681,0.727]	0.688 [0.661,0.712]	<0.05
**Philips D**	0.825 [0.804,0.844]	0.852 [0.834,0.868]	<0.05	0.679 [0.646,0.710]	0.692 [0.660,0.720]	<0.05

Comparison of body composition correlations between the body composition volume indices normalized by square of the height and the approximated anthropometric body composition indices before and after FOV extension at timepoint T0. FOV extension is performed on the images harmonized to the reference Siemens B50f kernel. The approximations were derived from the height and weight of the subject at baseline. We report the spearman rank correlations with 95% confidence intervals. Significance before and after FOV extension was evaluated using the method implemented by Hittner et al [38]. FM = Fat mass, FFM = Fat free mass, SAT = Subcutaneous adipose tissue.

**Fig 11:**
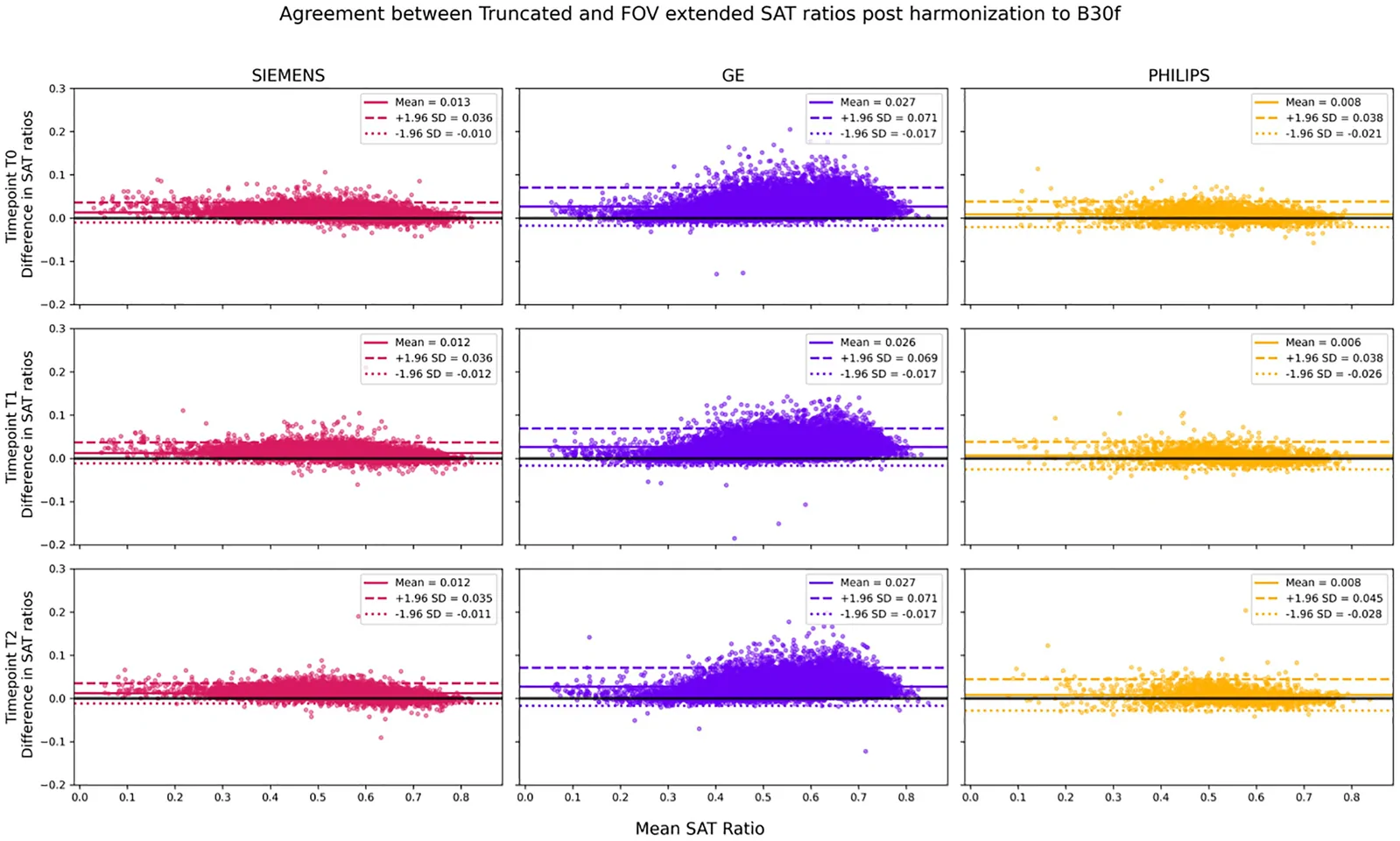
Bland Altman style plots for SAT ratios post harmonization to Siemens B30f and FOV extension. The amount of subcutaneous adipose tissue truncated depends on the field of view across different vendors. We use Bland Altman plots to compare the SAT ratios between truncated (unharmonized) and detruncated (harmonized to Siemens B30f) images across different vendors and timepoints, highlighting the mean difference and limits of agreement. Across all vendors and timepoints, we observe a consistent positive mean difference, indicating recovery of truncated SAT. The magnitude of recovery varies across vendors, reflecting vendor specific truncation patterns and the extent of missing tissue. The spread of differences remains consistent across all timepoints and vendors, indicating a systematic shift in the ratios.

**Fig 12:**
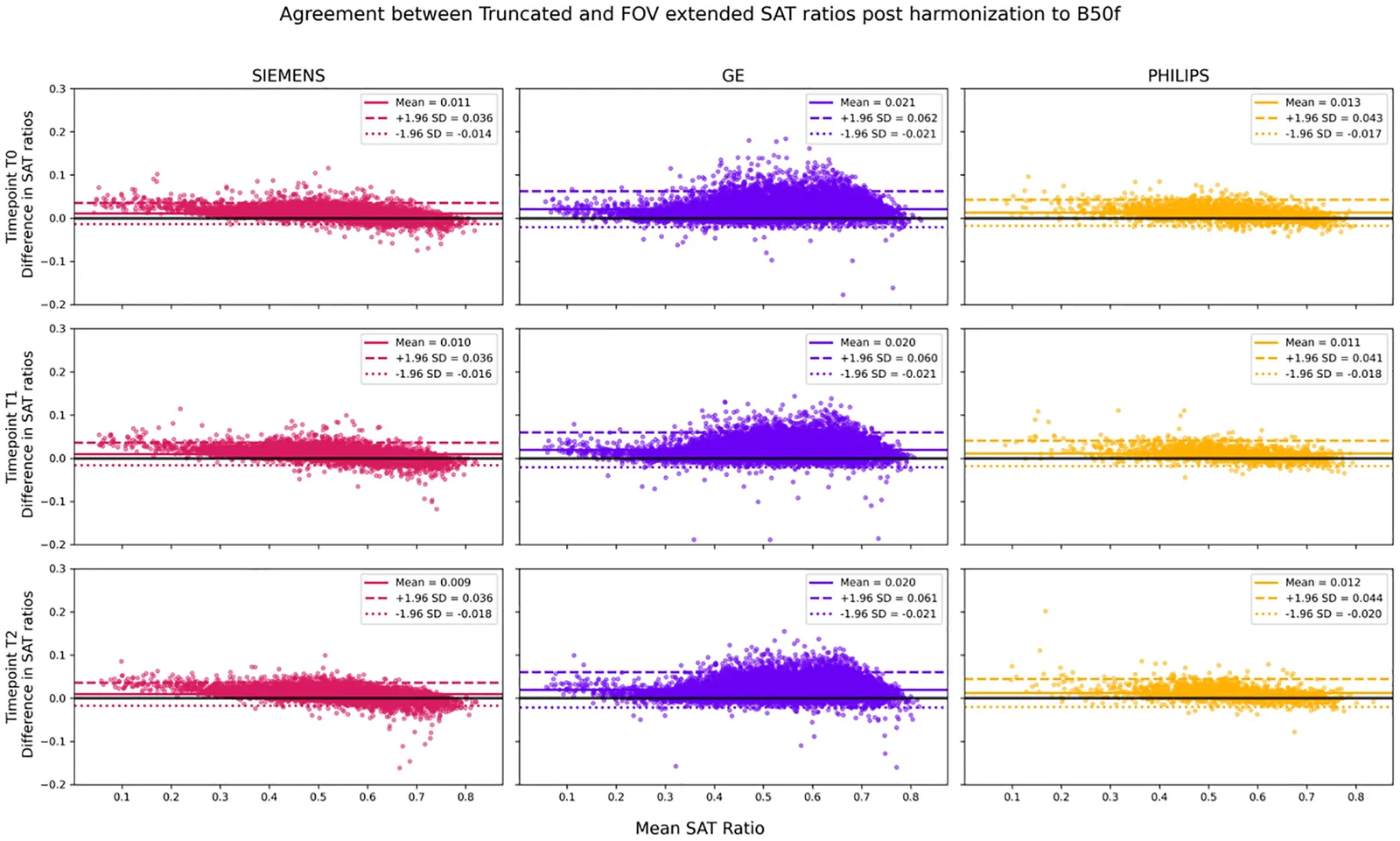
Bland Altman style plots for SAT ratios post harmonization to Siemens B50f and FOV extension. We present Bland Altman plots that compare SAT ratios between the truncated (unharmonized) images and detruncated (harmonized to Siemens B50f) across all vendors and timepoints. We observe an upward shift in the mean difference across all timepoints and vendors, suggesting the imputation of missing SAT regions that were removed due to truncation. However, the magnitude of the mean difference varies across vendors, highlighting the variability in truncation severity across different vendors.

### 3.3 Longitudinal analysis

Using the linear mixed effects model described in Section 2.4, we investigate the within subject trajectories for percent emphysema and SAT index. We report the fixed and random effect coefficients in Table 8, along with the ICC before and after harmonization. Prior to harmonization to the B30f soft kernel, percent emphysema measures exhibited strong kernel dependent effects. Hard kernels, including Siemens B50f, Siemens B80f, GE BONE, GE LUNG and Philips D showed large positive coefficients relative to the reference while soft kernels (GE STANDARD, Philips C and Philips B) showed negative coefficients. Following harmonization, the magnitude of the kernel coefficients reduced substantially. Covariates including age, sex and smoking status remained significant and beta coefficients for timepoint indicated progression across longitudinal visits. Subject level variance reflects the between-subject variability captured by the random intercept term. The subject level variance increased from 32.468 to 49.003 while the residual variance decreased from 7.245 to 6.227, resulting in an increase in the ICC from 81.8% to 88.8%. The increase in the subject level variance suggests that a greater proportion of the remaining variability was attributed to subject-specific differences rather than kernel bias. Similarly, before harmonization to the B50f hard kernel, kernel effect influenced emphysema measures, with GE BONE exhibiting negative coefficient and the remaining hard kernels showing positive coefficients relative to the reference. All soft kernels displayed negative coefficients. Harmonization minimized the magnitude of kernel coefficients with age, sex and smoking status remaining significant. Coefficients for longitudinal timepoints increased post harmonization. The within subject variance increased from 32.478 to 40.230 while the residual variance increased from 7.239 to 8.013, leading to an increase in ICC from 81.7% to 83.4%.

**Table 8:** Beta coefficients for emphysema and SAT index obtained from the linear mixed effects model.

Reference kernel for harmonization	Covariate	Coefficients for percent emphysema		Coefficients for body composition (SAT index)	
		Before	After	Before	After
**Siemens B30f**	**Intercept**	−2.405	−5.053	1706.162	1976.650
	**Timepoint T1**	0.173	0.109	25.256	33.268
	**Timepoint T2**	0.675	0.585	31.931	50.872
	**GE BONE**	4.853	1.850	−310.115	−40.187
	**GE LUNG**	14.775	1.826	−407.871	−64.212
	**GE STANDARD**	−3.705	3.269	−199.423	−18.299
	**Philips B**	−0.588	0.280	−160.637	−163.003
	**Philips C**	−2.030	−0.289	−101.336	−138.049
	**Philips D**	12.298	3.751	−217.504	−118.367
	**Siemens B50f**	11.719	1.201	−60.853	4.186
	**Siemens B80f**	28.363	2.297	−152.766	−24.422
	**Age**	0.187	0.240	−1.304	−3.149
	**Sex**	−1.942	−2.581	470.610	577.328
	**Smoking status**	−1.997	−2.391	−224.330	−287.101
	**Subject variance**	32.468	49.003	385622.259	563629.330
	**Residual variance**	7.245	6.227	50314.728	39728.495
**Siemens B50f**	**Intercept**	9.317	10.951	1643.516	1873.247
	**Timepoint T1**	0.176	0.325	25.281	30.513
	**Timepoint T2**	0.679	0.891	31.541	44.057
	**GE BONE**	−6.867	−1.323	−249.762	−34.567
	**GE LUNG**	3.053	0.713	−347.869	−80.542
	**GE STANDARD**	−15.426	−0.961	−138.366	−29.450
	**Philips B**	−12.388	−1.036	−97.666	−101.397
	**Philips C**	−13.759	−1.529	−42.701	−79.603
	**Philips D**	0.609	−2.521	−156.836	−81.058
	**Siemens B30f**	−11.720	−1.264	60.811	21.214
	**Siemens B80f**	16.644	3.231	−91.942	4.404
	**Age**	0.187	0.181	−1.271	−2.528
	**Sex**	−1.939	−2.855	470.316	582.188
	**Smoking status**	−2.000	−3.262	−224.103	−269.492
	**Subject variance**	32.478	40.230	385552.373	496848.046
	**Residual variance**	7.239	8.013	50563.350	36838.351

We report fixed-effect coefficients and subject-level random effects from linear mixed-effect models. For percent emphysema, coefficients are presented for ages before and after harmonization to the corresponding reference kernels. For body composition, SAT index coefficients are reported for the raw, truncated images (before) and harmonized, semantically extended images (after).

Since FOV extension was performed on images harmonized to their respective reference kernels, the reference kernel category was retained in the mixed effects model. For SAT index, kernel coefficients represent acquisition related offsets as opposed to biological differences. Compared to percent emphysema, both subject level and residual variances were larger, reflecting the higher magnitude of SAT index values derived from volumetric measures. Before FOV extension, SAT index measurements on truncated scans showed negative coefficients, suggesting that truncation patterns underestimate SAT measures. The largest deviations were observed for GE LUNG, GE BONE and Philips D. Following harmonization and FOV extension, the magnitude of kernel coefficients decreased across all kernels. Age was significant post harmonization while sex and smoking status continued to remain significant. The subject level variance increased from 385622.259 to 563629.330 while the residual variance decreased from 50314.728 to 39728.495, leading to an increase in ICC from 88.5% to 93.4%. The increase in the subject level variance attributes to the subject-specific differences that explain the remaining variability. We observe similar trends for all covariates associated with images that were semantically extended post harmonization to the Siemens B50f hard kernel. The subject level variance improved from 385552.373 to 496848.046 and the residual variance decreased from 50563.350 to 36838.351, resulting in an increase in the ICC from 88.4% to 93.1%.

### 3.4 Qualitative results

For paired reconstruction kernels, we inspect the texture of the lung parenchyma region across all the kernel pairs at timepoint T0. We select subjects from the 75^th^ percentile for each kernel pair where each subject exhibited a substantial amount of emphysema. Images reconstructed with hard and soft kernels exhibit a one to one pixel correspondence. However, the choice of kernel impacts the texture of the lung parenchyma that can further influence emphysema measures. Harmonization to the corresponding soft kernel within a given vendor enforces consistent texture in the lung parenchyma, enabling consistent and reproducible emphysema measures (Fig 13).

**Fig 13:**
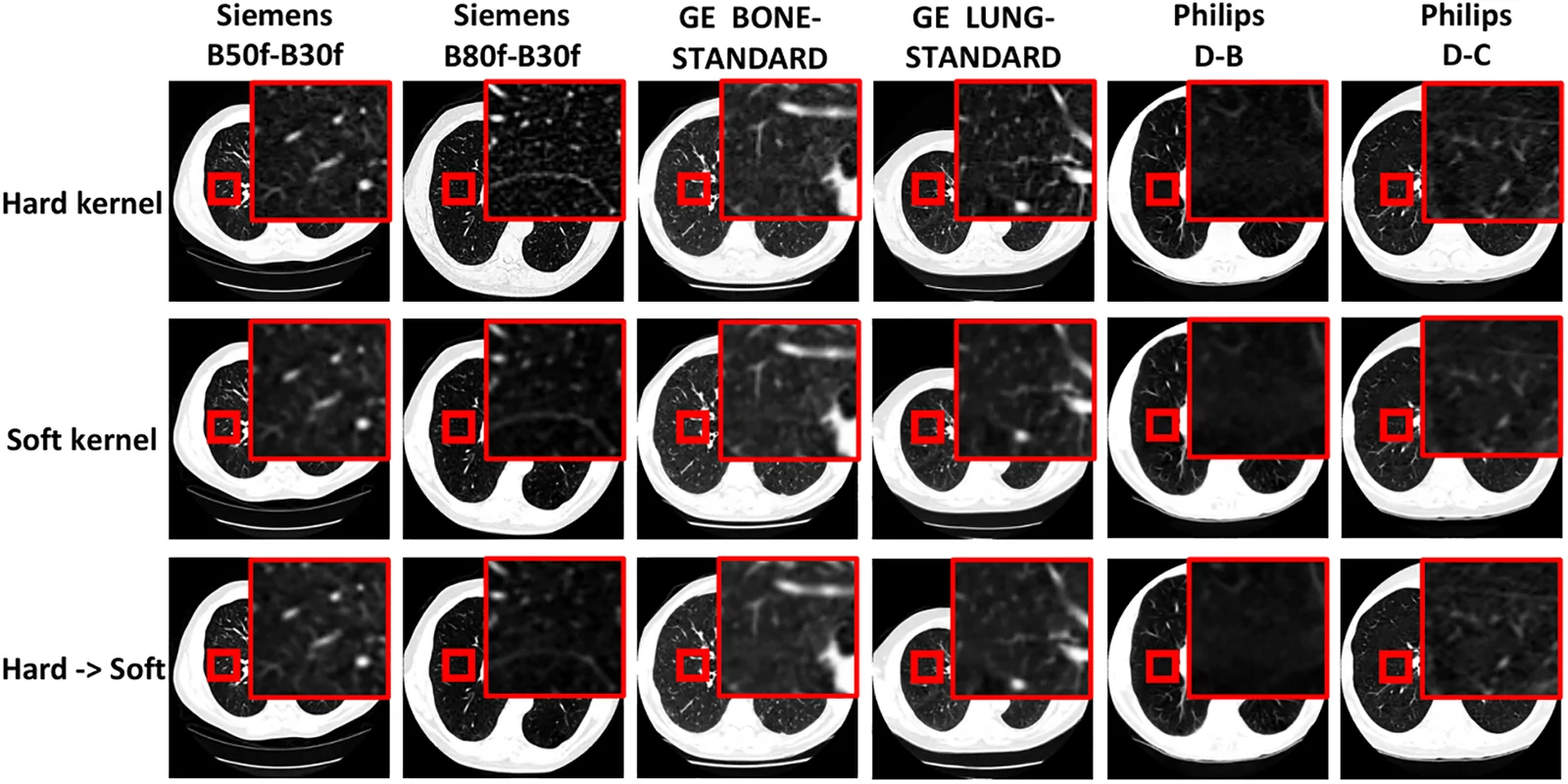
Qualitative inspection of lung parenchyma for paired kernels before and after harmonization. For a given subject, images reconstructed with a hard kernel and a corresponding soft kernel exhibit a one to one pixel correspondence with difference in the texture of the underlying anatomy due to the amount of pixel noise. Hard kernels enhance the texture while soft kernels smoothen the images. Harmonization to the corresponding soft kernel for a given pair ensures consistency in the texture of the underlying anatomy, enabling consistent and reproducible quantitative measurements.

At the cross sectional level, we present images obtained from timepoint T0 for subjects having prominent emphysematous regions. Images reconstructed with kernels obtained from different vendors exhibit varying degrees of sharpness and smoothness followed by positional differences in the way subjects were scanned. These images lack a one-to-one pixel correspondence, resulting in unpaired reconstruction kernels where a there is a lack of ground truth. Using our multi-domain kernel harmonization model, the shared latent space enables harmonization across unpaired kernels to the style of the reference kernel. Harmonization to the reference Siemens B30f soft kernel enforces a smooth texture in the lung parenchyma and surrounding regions (Fig 14) while harmonization to the reference Siemens B50f hard kernel leads to a sharper texture of the underlying anatomy (Fig 15).

**Fig 14:**
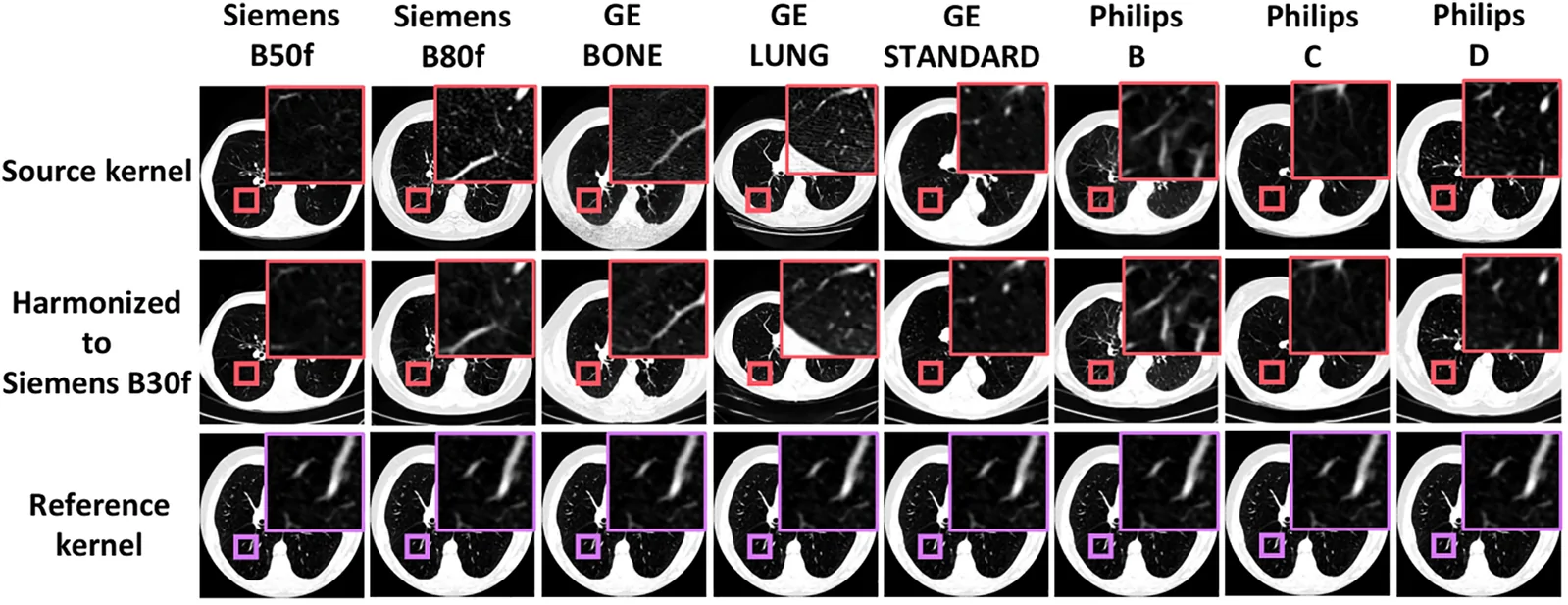
Qualitative inspection of lung parenchyma before and after harmonization to a reference soft kernel. Images reconstructed from kernels obtained from different vendors exhibit varying levels of sharpness and smoothness that can alter quantitative image measures derived from the underlying anatomy. Harmonization of all kernels to a reference Siemens B30f soft kernel ensures consistent texture of the underlying anatomy, benefitting quantitative image analysis that include percent emphysema and body composition.

**Fig 15:**
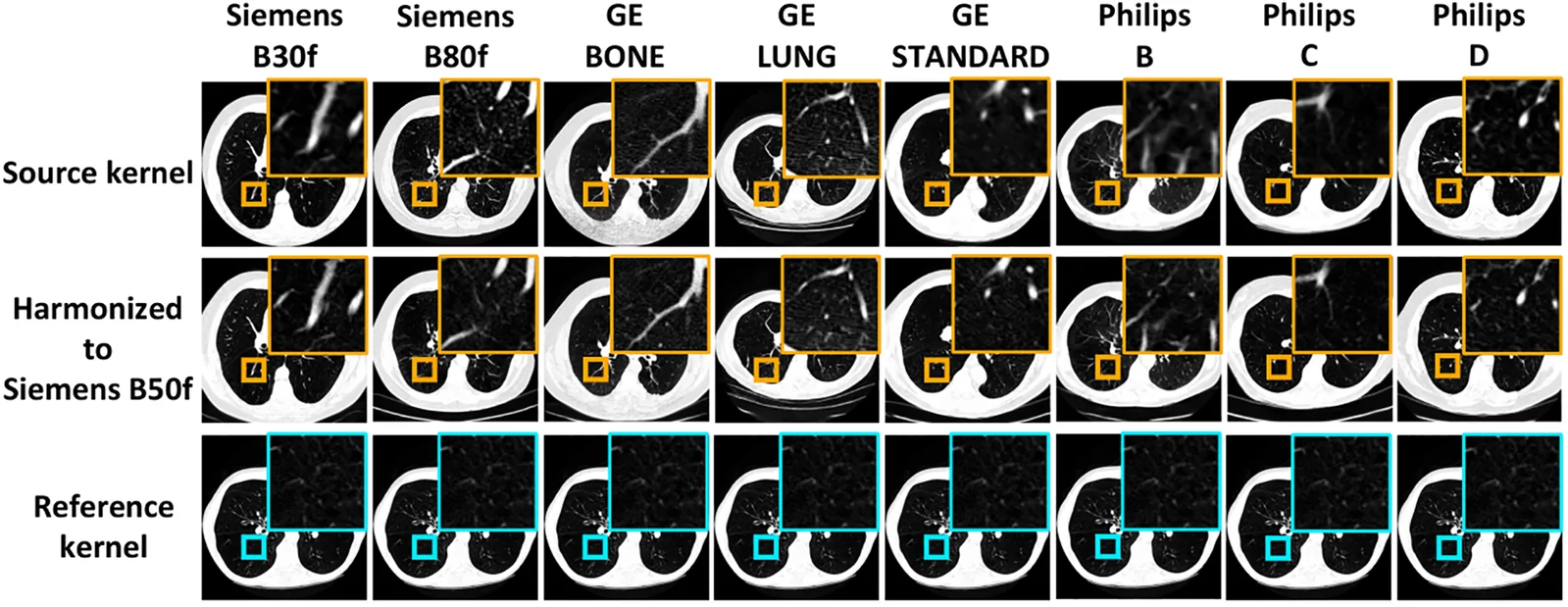
Qualitative inspection of lung parenchyma before and after harmonization to a reference hard kernel. We harmonize all images reconstructed with different kernels to a reference Siemens B50f hard kernel. For soft kernel images, we observe smooth texture in the lung parenchyma compared to the hard kernel images where the lung parenchyma is sharp. Harmonization to the style of the reference hard kernel, enforces a sharp texture in the highlighted region of interest for soft kernels while the degree of sharpness is modified to match the reference for the hard kernels.

Images reconstructed from the Siemens, GE and Philips vendors in lung screening CT exhibit different DFOV patterns that truncate peripheral skeletal muscle and SAT regions. The lack of missing tissue in the peripheral regions leads to an incomplete assessment of body composition measures. We present axial slices at the T5 vertebral level, determined by TotalSegmentator [26], that captures different extents of truncation for images reconstructed from the Siemens, Philips and GE vendors before harmonization and semantic extension (Figs 16 and 17). The axial slice was chosen based on the largest cross sectional area across all slices covered by the vertebrate level. Post harmonization, FOV extension imputes missing skeletal muscle and SAT regions that provide a complete extent for body composition assessment. However, there is a difference in the texture between the existing and imputed regions. Using TotalSegmentator [26], we verify the anatomical plausibility of the imputed regions using the tissue types task that generates segmentations for skeletal muscle and SAT regions. The imputed regions across all images are correctly identified as skeletal muscle (blue) and SAT (red). We present FOV extension for the remaining kernels across all vendors in the supplementary material (S3 and S4 Fig).

**Fig 16:**
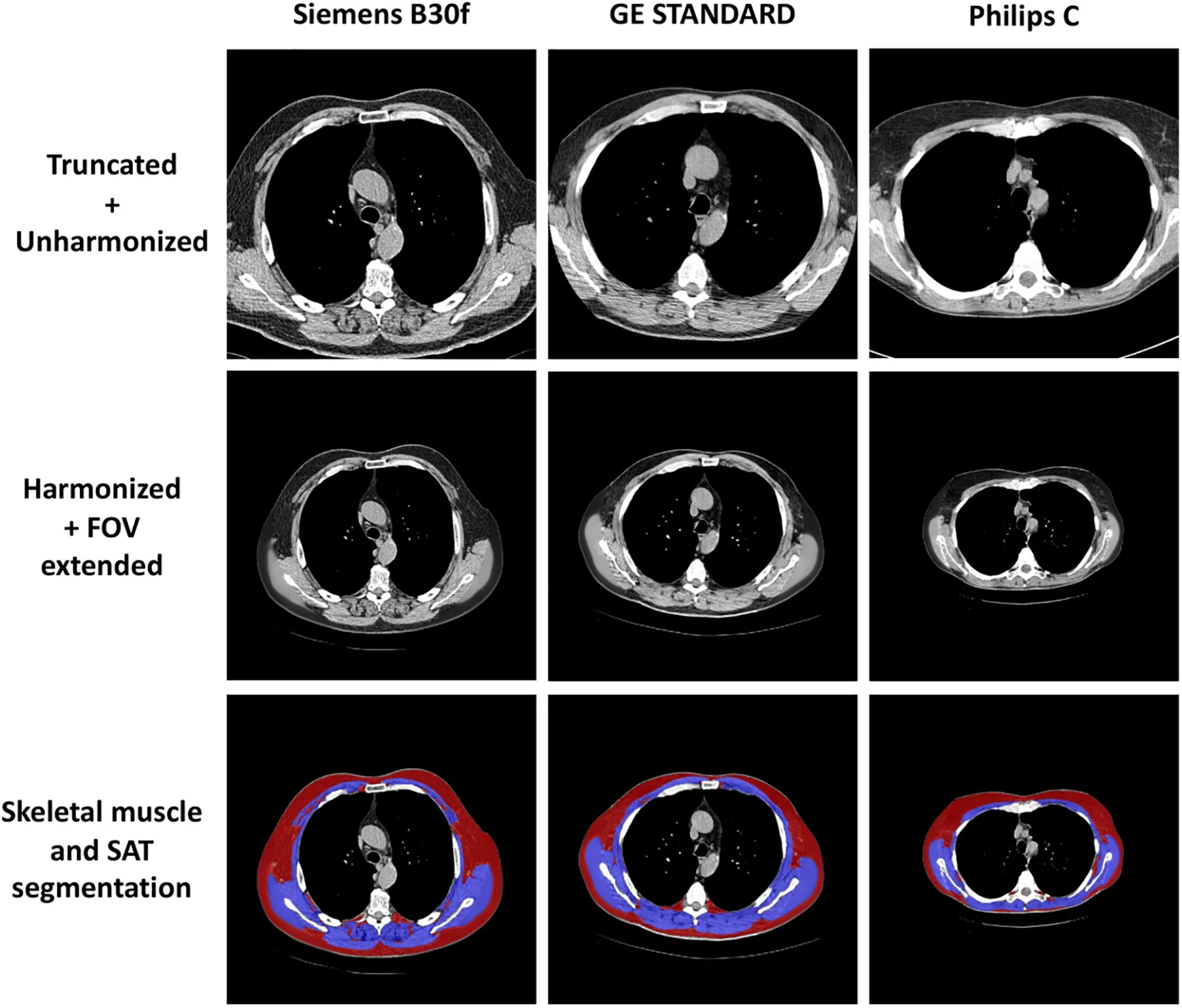
Qualitative analysis of FOV extension for kernels harmonized to Siemens B30f soft kernel. Vendor-specific truncation patterns in the NLST lead to incomplete skeletal muscle and SAT regions that limit whole body composition assessment. The extent of truncation across Siemens, GE and Philips vendors is presented for axial slices at the T5 vertebral level, windowed between [−150, 150] Hounsfield units. All images are harmonized to the Siemens B30f soft kernel and semantically extended, restoring peripheral SAT and skeletal muscle. Although imputed regions lack kernel texture, segmentation of muscle (blue) and SAT (red) remains anatomically consistent across truncated and imputed regions as seen from tissue types mask generated by TotalSegmentator.

**Fig 17:**
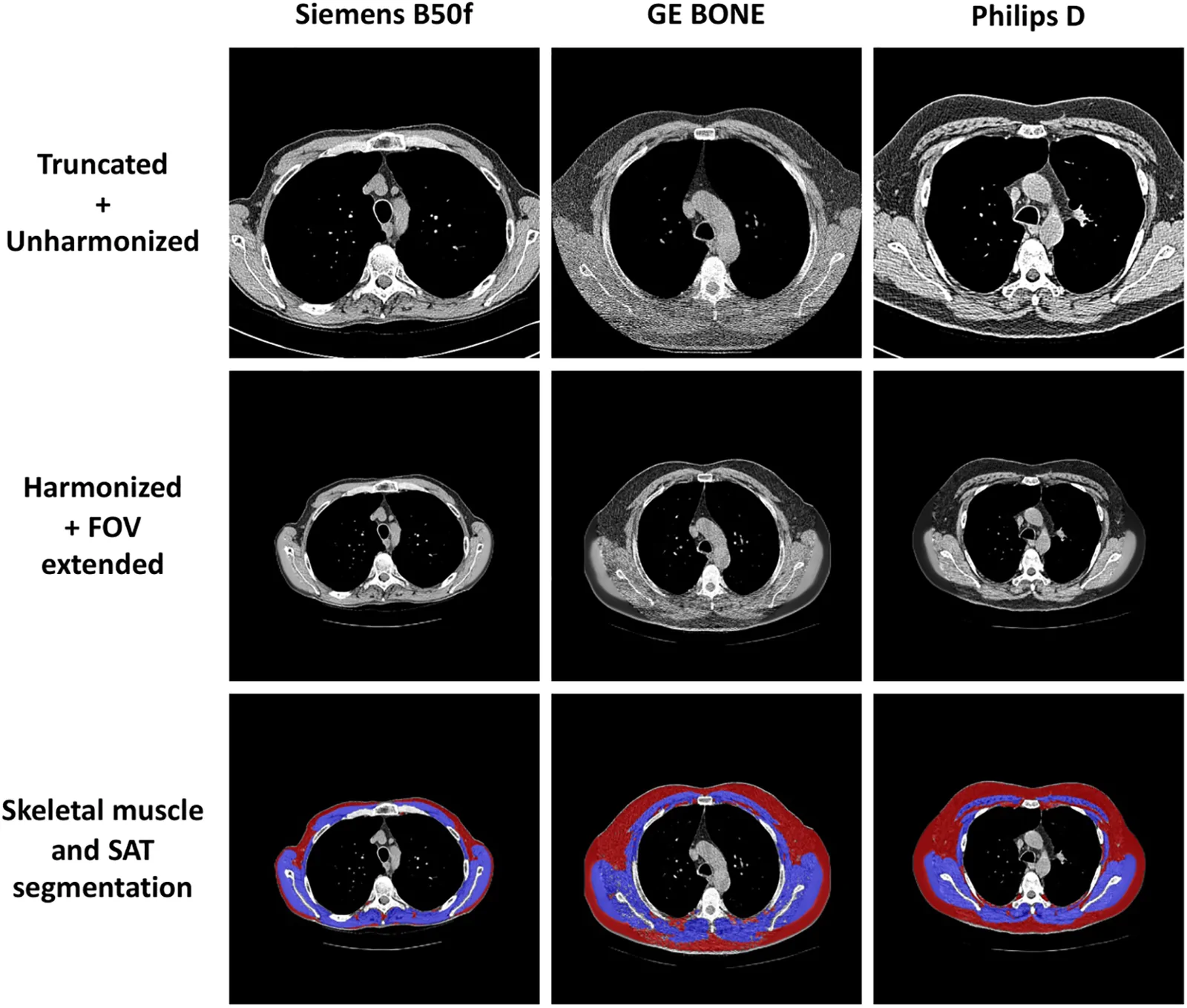
Qualitative analysis of FOV extension for kernels harmonized to Siemens B50f hard kernel. We present truncation patterns obtained from images reconstructed from hard kernel images obtained from the Siemens, GE and Philips vendors. All images are displayed at the T5 vertebral level and windowed between [−150, 150] Hounsfield units. The images are harmonized to the Siemens B50f hard kernel followed by semantic extension. FOV extension recovers missing skeletal muscle and SAT tissue that is anatomically consistent as seen from tissue types mask generated from TotalSegmentator. However, we see a difference in texture between the imputed and existing regions.

## 4 Discussion and conclusion

In this work, we deployed and validated a multi-stage standardization pipeline for low-dose lung cancer screening scans from the NLST. The curated dataset consists of scans reconstructed from nine reconstruction kernels across three vendors, exhibiting variability in through-plane resolution and field of view. Our pipeline standardizes through plane resolution followed by multi-domain kernel harmonization using an anatomically guided multipath cycleGAN, enabling intra-vendor and inter-vendor kernel harmonization within a unified model. Subsequently, harmonized images undergo semantic FOV extension, enabling comprehensive body composition assessment of skeletal muscle and SAT. We evaluated harmonization and FOV extension at paired, cross sectional and longitudinal levels using percent emphysema and body composition measures. The proposed pipeline reduced variability in emphysema and body composition measures at the intra-vendor and inter-vendor levels. Longitudinally, emphysema and body composition measures showed stability as evidenced by improved ICC driven by a reduction in residual variance and increase in between subject variability.

We investigated the efficacy of our proposed standardization pipeline across intra-vendor, inter-vendor and longitudinal data settings in the NLST. At the intra-vendor setting, subjects have scans reconstructed with a hard kernel and corresponding soft kernel, where a one-to-one pixel correspondence exists. However, hard kernels amplify the voxel noise relative to soft kernels. This is observed from the difference in the texture of the underlying anatomy, resulting in discrepancies in image similarity metrics within the lung, skeletal muscle and SAT regions. Harmonization to the reference soft kernel enforces consistent textures in the underlying anatomy, resulting in consistent improvements in RMSE, PSNR and SSIM. The performance of our proposed approach was compared to a histogram matching baseline to address the model complexity. Histogram matching corrects intensity differences by directly mapping one image intensity distribution onto the other. On the same kernel pairs from Siemens and GE vendors, our approach outperforms the baseline owing to the shared latent space that can learn a common representation across images followed by the adversarial nature of training that pushes the synthetic images close to the target than a distribution level correction. On the Philips pairs, histogram matching does better on RMSE and PSNR compared to our approach. This could be attributed to the complexity of the two-stage approach that focuses on adapting additional kernels to existing ones. However, our approach preserves structural detail as evidenced from the SSIM measures for lung and SAT regions. Additionally, differences in textures have implications on quantitative image measures. Hard kernels are known to overestimate emphysema compared to soft kernels [39] as a result of the amplified voxel noise. Harmonization mitigates these differences, enabling consistent and reproducible measures across all kernel pairs and timepoints. For body composition measures, differences in skeletal muscle and SAT volumes suggest inconsistencies attributed to kernel, with notable underestimation of SAT volumes. Harmonization mitigates discrepancies in body composition measures, ensuring consistency across all pairs. Overall, these findings are consistent with prior studies that investigate paired kernel harmonization through emphysema and body composition measures [19,24,40], demonstrating the ability of our unified standardization pipeline to produce consistent and reproducible measures at the intra-vendor level.

While paired intra-vendor analysis presents a reference soft kernel for each subject that verifies harmonization, cross sectional settings acquire scans across multiple subjects, vendors and reconstruction settings. Scans exhibit variability in spatial resolution, field of view and reconstruction kernel across vendors, leading to a lack of one-to-one correspondence between scans. The variability in reconstruction kernels impact percent emphysema quantification while truncation patterns hinder the ability to attain whole body composition measures. We therefore evaluated the proposed standardization pipeline across all cross sectional timepoints in the NLST, assessing the ability to obtain consistent percent emphysema measures and body composition measures for the whole extent of the body. Emphysema distributions vary across kernels with hard kernels showing overestimation compared to soft kernels. Harmonization to the reference soft and hard kernels mitigated bias in measures as evidenced from the small to medium effect sizes, suggesting improved consistency in measures. Field of view extension led to increased SAT ratios as evidenced from the positive mean difference on the Bland Altman plots, suggesting imputation of peripheral SAT. Furthermore, the improved correlations of muscle and SAT indices with anthropometric measures suggest that body composition measures are more consistent post FOV extension. The improvements in SAT and muscle correlations can be attributed to FOV extension rather than kernel harmonization, that has already been validated on a subset of images from the NLST by Xu et.al [20]. Particularly, the interpretations are consistent with larger gains observed for GE images, which exhibited more pronounced FOV truncation in unharmonized images. Overall our results result are consistent with previous works [20] with FOV extension having also added value to lung cancer diagnosis [12].

Across longitudinal timepoints, subjects may acquire scans with different reconstruction kernels from different vendors that can obscure the true biological progression due to acquisition related variability. We assessed the impact of our proposed pipeline in the longitudinal setting across all scans and investigated the stability of biomarkers and preservation of within subject variability. On the unharmonized images, kernel bias dominated emphysema measures with hard kernels showing higher coefficients compared to the reference soft kernel with soft kernels underestimating these measures. The increase in magnitude in the timepoint beta coefficients suggested changes over time when compared to the baseline T0. Post harmonization to the reference B30f soft kernel, kernel coefficients reduced, suggesting the mitigation of bias in emphysema measures. Additionally, an increase in the between subject variance and decrease in the within subject variance lead to a higher ICC, suggesting that emphysema measures were more stable across time. While harmonization to the B50f kernel mitigated kernel bias and showed improvements in the between subject variance, the within subject variance increased. This can be attributed to the overestimation of emphysema when harmonized to a hard kernel. Therefore, it is advisable to choose a soft kernel reference for longitudinal analysis. In the case of body composition measures, SAT indices were underestimated due to truncation across longitudinal timepoints which is reflected in the kernel coefficients. Post harmonization to any reference, kernel coefficients are minimized followed by improvements in the ICC. These results suggest that SAT indices are dependent on the truncation pattern of the corresponding vendor and semantic extension promotes whole body composition assessment. We note that residual kernel associated differences remained after harmonization, particularly for Siemens B80f and GE LUNG. Their clinical significance is unknown since outcome specific thresholds for meaningful change over comparable longitudinal intervals were not evaluated. Furthermore, when reconstruction kernels differ across visits, residual measurement differences may affect estimates of longitudinal progression despite statistical adjustment for kernel. Further validation is needed to determine the influence of these residual effects on longitudinal analyses.

Our work is not without its limitations. The NLST images were reconstructed using filtered back projection (FBP) which was the standard reconstruction method available during the trial. In contrast, modern LDCT increasingly uses iterative and deep learning reconstruction techniques, that can produce non-linear spatially varying noise characteristics that differ from those of FBP [41,42]. Since our harmonization framework learns transformations between the noise textures of kernels using FBP, the trained pipeline is specific to FBP reconstructed images, requiring additional fine tuning or retraining before application to images reconstructed with modern techniques. Extending the framework to accommodate these reconstruction techniques represents an important direction for future work. Although the final harmonized cohort remained broadly representative of the full NLST cohort, differences in the racial distribution of participants with Toshiba or underrepresented-kernel scans (Supplementary Table **ST2** in S1 File) may limit the generalizability of our findings to demographic groups and acquisition sites disproportionately associated with these excluded imaging conditions. For the images reconstructed from the Philips vendors, we occasionally notice hallucinations in the peripheral SAT regions for images harmonized to the reference Siemens B30f kernel (S5 Fig). This could be attributed to the instability in the stage two training of the multi-domain kernel harmonization model where the goal is to adapt reconstruction kernels from a new vendor to existing vendors. Similarly, performance for the (B, D) and (C, D) pairs from the Philips vendor fell short in comparison to the other vendors. The reduced sampling exposure, combined with the difficulty of aligning the Philips kernels to the existing representation may have contributed to the weaker performance. These limitations could be addressed through improved training strategies that involve the utilization of a unique kernel from each vendor during stage one training. We observed minor improvements in quantitative image measures at longitudinal timepoints for certain kernels as opposed to the baseline timepoint due to our training data consisting of images obtained at timepoint T0. Incorporating longitudinal data during training could benefit quantitative imaging at the cross sectional and longitudinal levels. We occasionally observed artifacts in the GE images harmonized to the Siemens B30f kernel in the peripheral muscle and SAT regions. However, these artifacts did not hamper the downstream body composition analysis. Furthermore, the NLST accounts for the weight of the subject during initial screening only. The correlations of the muscle and SAT indices at longitudinal timepoints were obtained using the baseline weight measurements serving as a proxy, which may not indicate the true biological correlation at timepoints T1 and T2.

In conclusion, we developed a unified standardization pipeline to address heterogeneity in reconstruction kernels, axial spatial resolution and field of view in low-dose chest CT scans acquired in the NLST. By integrating through plane resampling, multi-domain kernel harmonization and semantic field of view extension, our approach mitigates acquisition variability, providing a consistent representation across diverse image settings. We demonstrate that the proposed pipeline reduces kernel and field of view heterogeneity in intra-vendor, cross sectional and longitudinal settings, enabling consistent and reproducible quantitative imaging measures. We aim to publicly release the harmonized NLST dataset following data return to the NLST, highlighting its broader impact for the lung cancer research community.

## Supporting information

S1 File Supplementary tables. (DOCX)

S1 Fig Rare cases of image artifacts excluded during quality assurance. We present rare instances of images that exhibited severe artifacts in the NLST. Images that displayed a ruptured breast implant, severe artifact in the vertebrates due to metal implant, cupping artifact and removal of lung lobe resulting in irregular lung anatomy were excluded during quality assurance.
(TIFF)

S2 Fig Two-stage multipath cycleGAN for multi-domain kernel harmonization. Inter-vendor and intra-vendor harmonization can be performed across various kernels using an anatomically guided multipath cycleGAN through a shared latent space “L” using a two-stage approach. Stage one training focuses on harmonizing kernels obtained from the Siemens and GE manufacturers using domain specific encoders, decoders and discriminators. In stage two, we introduce images reconstructed with the Philips kernels and harmonize across all possible combinations of stage one and stage two domains.
(TIFF)

S3 Fig Field of view extension for images harmonized to Siemens B30f soft kernel. We present the axial slices at the T5 vertebral level for the remaining kernels obtained from the Siemens, GE and Philips vendors. Truncation patterns are vendor dependent, leading to incomplete muscle and SAT regions. All images are harmonized to the Siemens B30f soft kernel, followed by semantic field of view extension. The anatomical consistency of muscle (red) and SAT (blue) is verified by the tissue types mask from TotalSegmentator. However, the texture of the imputed regions differs from the existing muscle and SAT regions.
(TIFF)

S4 Fig Field of view extension for images harmonized to the Siemens B50f hard kernel. We present the axial slices at the T5 vertebral level for the remaining kernels obtained from the Siemens, GE and Philips vendors. Truncation patterns are vendor dependent, leading to incomplete muscle and SAT regions. All images are harmonized to the Siemens B50f hard kernel, followed by semantic field of view extension. The anatomical consistency of muscle (red) and SAT (blue) is verified by the tissue types mask from TotalSegmentator. However, the texture of the imputed regions differs from the existing muscle and SAT regions.
(TIFF)

S5 Fig Failure cases for the Philips kernel images harmonized to the Siemens B30f soft kernel. We occasionally observe hallucinations in images reconstructed with Philips kernels post harmonization to the Siemens B30f kernel. For the Philips D image, a minor hallucination (black hole) exists at the periphery of SAT, distorting the anatomy. For the Philips B image, we observe a failure case where skeletal muscle regions close to the lung are misclassified as the lung region.
(TIFF)

## References

[1] Naidich DP, Marshall CH, Gribbin C, Arams RS, Mccauley DI. Low-Dose CT of the Lungs: Preliminary Observations. 10.1148/radiology.175.3.2343122 2343122

[2] Henschke CI, McCauley DI, Yankelevitz DF, Naidich DP, McGuinness G, Miettinen OS, et al. Early Lung Cancer Action Project: Overall Design and Findings from Baseline Screening. Lancet. 1999;354(9173):99–105. doi: 10.1016/S0140-6736(99)06093-6 10408484

[3] Reduced lung-cancer mortality with low-dose computed tomographic screening. N Engl J Med. 2011;365(5):395–409. doi: 10.1056/NEJMoa1102873 PMC4356534 21714641

[4] Gierada DS, Black WC, Chiles C, Pinsky PF, Yankelevitz DF. Low-Dose CT Screening for Lung Cancer: Evidence from 2 Decades of Study. Radiol Imaging Cancer. 2020;2(2):e190058. doi: 10.1148/rycan.2020190058 32300760 PMC7135238

[5] Clark K, Vendt B, Smith K, Freymann J, Kirby J, Koppel P, et al. The Cancer Imaging Archive (TCIA): maintaining and operating a public information repository. J Digit Imaging. 2013;26(6):1045–57. doi: 10.1007/s10278-013-9622-7 23884657 PMC3824915

[6] Mikhael PG, Wohlwend J, Yala A, Karstens L, Xiang J, Takigami AK, et al. Sybil: A Validated Deep Learning Model to Predict Future Lung Cancer Risk From a Single Low-Dose Chest Computed Tomography. J Clin Oncol. 2023;41(12):2191–200. doi: 10.1200/JCO.22.01345 36634294 PMC10419602

[7] Li TZ, Xu K, Gao R, Tang Y, Lasko TA, Maldonado F, et al. Time-distance vision transformers in lung cancer diagnosis from longitudinal computed tomography. J Med Imaging. 2023;12(4):229–38. doi: 10.1117/12.2653911 PMC10353776 37465096

[8] Gao R, Tang Y, Khan MS, Xu K, Paulson AB, Sullivan S. Cancer risk estimation combining lung screening ct with clinical data elements. Radiol Artif Intell. 2021;3(6). doi: 10.1148/ryai.2021210032 PMC8637232 34870220

[9] Li TZ, Zuo L, Liu Y, Krishnan AR, Sandler KL, Lasko TA, et al. Contrastive Patient-level Pretraining Enables Longitudinal and Multimodal Fusion for Lung Cancer Risk Prediction. Proc Mach Learn Res. 2025;301:1008–20. 42180648 PMC13197062

[10] Li TZ, Lee HH, Xu K, Gao R, Dawant BM, Maldonado F, et al. Quantifying emphysema in lung screening computed tomography with robust automated lobe segmentation. J Med Imaging. 2023;10(4):044002. doi: 10.1117/1.JMI.10.4.044002 PMC10353481 37469854

[11] Chiles C, Duan F, Gladish GW, Ravenel JG, Baginski SG, Snyder BS, et al. Association of Coronary Artery Calcification and Mortality in the National Lung Screening Trial: A Comparison of Three Scoring Methods. Radiology. 2015;276(1):82–90. doi: 10.1148/radiol.15142062 25759972 PMC5137795

[12] Xu K, Khan MS, Li TZ, Gao R, Terry JG, Huo Y. AI Body Composition in Lung Cancer Screening: Added Value Beyond Lung Cancer Detection. Radiology. 2023;308(1). doi: 10.1148/RADIOL.222937/SUPPL_FILE/RY222937SUPPF1B.JPG 37489991 PMC10374937

[13] Abdulkareem NK, Hajee SI, Hassan FF, Ibrahim IK, Al-Khalidi REH, Abdulqader NA. Investigating the slice thickness effect on noise and diagnostic content of single-source multi-slice computerized axial tomography. J Med Life. 2023;16(6):862–7. doi: 10.25122/jml-2022-0188 37675166 PMC10478658

[14] Kim H, Goo JM, Ohno Y, Kauczor H-U, Hoffman EA, Gee JC, et al. Effect of Reconstruction Parameters on the Quantitative Analysis of Chest Computed Tomography. J Thorac Imaging. 2019;34(2):92–102. doi: 10.1097/RTI.0000000000000389 30802233

[15] Xu Y, Lu L, Sun SH, E L-N, Lian W, Yang H, et al. Effect of CT image acquisition parameters on diagnostic performance of radiomics in predicting malignancy of pulmonary nodules of different sizes. Eur Radiol. 2022;32(3):1517–27. doi: 10.1007/s00330-021-08274-1 34549324 PMC9136691

[16] Gierada DS, Bierhals AJ, Choong CK, Bartel ST, Ritter JH, Das NA, et al. Effects of CT section thickness and reconstruction kernel on emphysema quantification relationship to the magnitude of the CT emphysema index. Acad Radiol. 2010;17(2):146–56. doi: 10.1016/j.acra.2009.08.007 19931472 PMC2818169

[17] Schaller S, Wildberger JE, Raupach R, Niethammer M, Klingenbeck-Regn K, Flohr T. Spatial domain filtering for fast modification of the tradeoff between image sharpness and pixel noise in computed tomography. IEEE Trans Med Imaging. 2003;22(7):846–53. doi: 10.1109/TMI.2003.815073 12906238

[18] Boedeker KL, McNitt-Gray MF, Rogers SR, Truong DA, Brown MS, Gjertson DW, et al. Emphysema: effect of reconstruction algorithm on CT imaging measures. Radiology. 2004;232(1):295–301. doi: 10.1148/radiol.2321030383 15220511

[19] Tanabe N, Kaji S, Shima H, Shiraishi Y, Maetani T, Oguma T, et al. Kernel Conversion for Robust Quantitative Measurements of Archived Chest Computed Tomography Using Deep Learning-Based Image-to-Image Translation. Front Artif Intell. 2022;4:769557. doi: 10.3389/frai.2021.769557 35112080 PMC8801695

[20] Xu K, Li T, Khan MS, Gao R, Antic SL, Huo Y, et al. Body composition assessment with limited field-of-view computed tomography: A semantic image extension perspective. Med Image Anal. 2023;88:102852. doi: 10.1016/j.media.2023.102852 37276799 PMC10527087

[21] Krishnan AR, Li TZ, Remedios LW, Kim ME, Gao C, Rudravaram G. Multipath cycleGAN for harmonization of paired and unpaired low-dose lung computed tomography reconstruction kernels. Med Phys. 2025;52(11). doi: 10.1002/MP.70120 41206352 PMC12596234

[22] Krishnan AR, Xu K, Li TZ, Remedios LW, Sandler KL, Maldonado F. Lung CT harmonization of paired reconstruction kernel images using generative adversarial networks. Med Phys. 2024. doi: 10.1002/mp.17028 PMC11321937 38530135

[23] Krishnan A, Li T, Remedios LW, Xu K, Zuo L, Sandler KL, et al. Anatomy-Guided Multi-Path CycleGAN for Lung CT Kernel Harmonization. Proceedings of Machine Learning Research [Internet]. 2025 [cited 2026 Feb 16]. p. 1–16. Available from: https://github.com/MASILab/AnatomyconstrainedMultipathGAN

[24] Lee SM, Lee JG, Lee G, Choe J, Do KH, Kim N, et al. CT Image Conversion among Different Reconstruction Kernels without a Sinogram by Using a Convolutional Neural Network. Korean J Radiol. 2019;20(2):295–303. doi: 10.3348/kjr.2018.0249 30672169 PMC6342751

[25] Xu K, Li T, Khan MS, Gao R, Antic SL, Huo Y, et al. Body Composition Assessment with Limited Field-of-view Computed Tomography: A Semantic Image Extension Perspective [Internet]. 2022. Available from: http://arxiv.org/abs/2207.06551 10.1016/j.media.2023.102852 PMC10527087 37276799

[26] Wasserthal J, Breit H-C, Meyer MT, Pradella M, Hinck D, Sauter AW, et al. TotalSegmentator: Robust Segmentation of 104 Anatomic Structures in CT Images. Radiol Artif Intell. 2023;5(5):e230024. doi: 10.1148/ryai.230024 37795137 PMC10546353

[27] Li X, Morgan PS, Ashburner J, Smith J, Rorden C. The first step for neuroimaging data analysis: DICOM to NIfTI conversion. J Neurosci Methods. 2016;264:47–56. doi: 10.1016/j.jneumeth.2016.03.001 26945974

[28] Andersen HK, Völgyes D, Martinsen ACT. Image quality with iterative reconstruction techniques in CT of the lungs-A phantom study. Eur J Radiol Open. 2018;5:35–40. doi: 10.1016/j.ejro.2018.02.002 29719856 PMC5926271

[29] Mackin D, Ger R, Gay S, Dodge C, Zhang L, Yang J, et al. Matching and Homogenizing Convolution Kernels for Quantitative Studies in Computed Tomography. Invest Radiol. 2019;54(5):288–95. doi: 10.1097/RLI.0000000000000540 30570504 PMC6449212

[30] Kim ME, Gao C, Newlin NR, Rudravaram G, Krishnan AR, Ramadass K, et al. Scalable quality control on processing of large diffusion-weighted and structural magnetic resonance imaging datasets. PLoS One. 2025;20(8):e0327388. doi: 10.1371/journal.pone.0327388 40748971 PMC12316263

[31] Krishnan AR, Xu K, Li TZ, Gao C, Remedios LW, Kanakaraj P, et al. Inter-vendor harmonization of CT reconstruction kernels using unpaired image translation. In. SPIE-Intl Soc Optical Eng. 2024. 45 p. 10.1117/12.3006608 PMC11392419 39268356

[32] Kingma DP, Lei Ba J. ADAM: A method for stochastic optimization.

[33] Mao X, Li Q, Xie H, Lau RYK, Wang Z, Smolley SP. Least Squares Generative Adversarial Networks. 10.1109/TPAMI.2018.2872043 30273144

[34] Wang Z, Gu S, Leader JK, Kundu S, Tedrow JR, Sciurba FC, et al. Optimal threshold in CT quantification of emphysema. Eur Radiol. 2013;23(4):975–84. doi: 10.1007/s00330-012-2683-z 23111815 PMC3573224

[35] Bland JM, Altman DG. Measurement statistical methods for assessing agreement between two methods of clinical measurement. Lancet. 1986;327(8476):307–10. 2868172

[36] Cohen J. Statistical Power Analysis for the Behavioral Sciences Second Edition.

[37] Bak SH, Kim JH, Jin H, Kwon SO, Kim B, Cha YK, et al. Emphysema quantification using low-dose computed tomography with deep learning-based kernel conversion comparison. Eur Radiol. 2020;30(12):6779–87. doi: 10.1007/s00330-020-07020-3 32601950

[38] Hittner JB, May K, Silver NC. A Monte Carlo evaluation of tests for comparing dependent correlations. J Gen Psychol. 2003;130(2):149–68. doi: 10.1080/00221300309601282 12773018

[39] Gallardo-Estrella L, Lynch DA, Prokop M, Stinson D, Zach J, Judy PF, et al. Normalizing computed tomography data reconstructed with different filter kernels: effect on emphysema quantification. Eur Radiol. 2016;26(2):478–86. doi: 10.1007/s00330-015-3824-y 26002132 PMC4712239

[40] Jin H, Heo C, Kim JH. Deep learning-enabled accurate normalization of reconstruction kernel effects on emphysema quantification in low-dose CT. Phys Med Biol. 2019;64(13):135010. doi: 10.1088/1361-6560/ab28a1 31185463

[41] Koetzier LR, Mastrodicasa D, Szczykutowicz TP, van der Werf NR, Wang AS, Sandfort V, et al. Deep learning image reconstruction for CT: technical principles and clinical prospects. Radiology. 2023;306(3). doi: 10.1148/RADIOL.221257 36719287 PMC9968777

[42] Mohammadinejad P, Mileto A, Yu L, Leng S, Guimaraes LS, Missert AD, et al. CT Noise-Reduction Methods for Lower-Dose Scanning: Strengths and Weaknesses of Iterative Reconstruction Algorithms and New Techniques. RadioGraphics. 2021;41(5):1493–508. doi: 10.1148/rg.2021200196 34469209

