# Supplementary material for: Harmonizing the National Lung Screening Trial: Kernel resolution and field of view compensation
Source: PLoS One. 2026 Oct 5;21(10):e0352075. doi: 10.1371/journal.pone.0352075 (PMC13637926; doi:10.1371/journal.pone.0352075)
Supplement: Supplementary file 1 [file pone.0352075.s001.docx]

**Supplementary tables**

**Table ST1. Reconstruction kernels excluded from the NLST analysis.**

| **Manufacturer** | **Kernel** | **No. of scans across all timepoints** |
| --- | --- | --- |
| **GE** | EXPERIMENTAL7  SOFT | 18573  3 |
| **PHILIPS** | A  EC | 163  5 |
| **TOSHIBA** | FC01  FC02  FC10  FC11  FC30  FC50  FC51  FC52  FC53  FC82  FL01  FL04 | 236  109  1384  361  383  55  4669  1  8  31  4873  30 |
| **SIEMENS** | T20s  B20f  B30s  B31f  B31s  B35f  B36f  B40f  B41s  B45f  B50s  B60f  B60s  B70f | 21423  40  28  32  14  2  1  4  1  16  7  140  18  127 |

We excluded a total of 30 kernels from the NLST. While EXPERIMENTAL7 and T20s have a large count, these kernels were exclusively used for localizer/scout scans. TOSHIBA scans were not included in the analysis while the remaining kernels from SIEMENS, GE and PHILIPS were excluded due to insufficient representation across all timepoints.

**Table ST2.** **Demographic information of included participants for harmonization, excluded kernel participants and the entire NSLT cohort before filtering**

| **Demographic** | **Full NLST cohort before filtering**  **(n=25206)** | **Final NLST cohort for analysis**  **(n=23669)** | **Cohort with excluded Toshiba and underrepresented kernels**  **(n=2519)** |
| --- | --- | --- | --- |
| **Age at baseline** | 61 (57-65) | 61 (57-65) | 60 (57-65) |
| **Sex** |  |  |  |
| **Male** | 14918 | 13919 | 1648 |
| **Female** | 10288 | 9750 | 871 |
| **Race** |  |  |  |
| **White** | 22968 | 22073 | 1835 |
| **Black or African-American** | 1101 | 1091 | 31 |
| **Asian** | 546 | 96 | 454 |
| **American Indian or Alaskan** | 85 | 82 | 5 |
| **Native Hawaiian or Other Pacific Islander** | 87 | 12 | 76 |
| **Multiple Race** | 321 | 218 | 110 |
| **Unknown** | 98 | 97 | 8 |
| **Smoking status** |  |  |  |
| **Former** | 13113 | 12282 | 1398 |
| **Current** | 12093 | 11387 | 1121 |
| **Pack years** | 48 (39-66.25) | 48 (39-66.5) | 48 (39-65) |
| **Lung cancer** |  |  |  |
| **No cancer** | 24146 | 22681 | 2424 |
| **Cancer** | 1060 | 988 | 95 |

We present the demographic information across three NLST cohorts: a) NLST cohort before filtering b) NSLT cohort included for the analysis and c) NLST cohort where Toshiba and underrepresented kernels were excluded. Age and pack years are represented as median and inter quartile range in brackets.

**Table ST3.** **Image similarity metrics obtained for paired kernels at timepoint T1.**

| **Region of interest** | **Kernel pairs** | **RMSE** | | **PSNR** | | **SSIM** | |
| --- | --- | --- | --- | --- | --- | --- | --- |
|  |  | **Before** | **After** | **Before** | **After** | **Before** | **After** |
| **Lung** | **(B30f, B50f)** | 44.88  (10.22) | 28.57  (4.88) | 27.15 (1.77) | 31.00 (1.37) | 0.73 (0.05) | 0.93  (0.02) |
|  | **(B30f, B80f)** | 108.92 (27.52) | 27.93  (4.79) | 19.48 (1.90) | 31.18 (1.26) | 0.51 (0.03) | 0.93  (0.03) |
|  | **(STANDARD,**  **BONE)** | 51.78  (17.72) | 36.22  (5.08) | 26.15 (2.69) | 28.91 (1.22) | 0.70  (0.70) | 0.90  (0.04) |
|  | **(STANDARD,**  **LUNG)** | 74.31  (27.52) | 46.42  (7.59) | 23.05 (2.73) | 26.77 (1.31) | 0.64 (0.07) | 0.87  (0.06) |
|  | **(C, D)** | 49.02  (10.85) | 43.67  (7.34) | 26.39 (1.85) | 27.31 (1.41) | 0.74 (0.05) | 0.84  (0.05) |
|  | **(B, D)** | 33.04  (9.74) | 35.12  (3.53) | 29.87 (1.88) | 29.13 (0.86) | 0.88 (0.08) | 0.89  (0.03) |
| **Skeletal muscle** | **(B30f, B50f)** | 27.00  (4.49) | 14.83  (2.68) | 16.55 (1.40) | 21.76 (1.46) | 0.71 (0.06) | 0.87  (0.03) |
|  | **(B30f, B80f)** | 45.65  (5.10) | 15.18  (3.35) | 11.92 (0.95) | 21.60 (1.61) | 0.55 (0.06) | 0.87  (0.03) |
|  | **(STANDARD,**  **BONE)** | 30.85  (6.35) | 17.48  (4.22) | 15.45 (1.77) | 20.44 (1.97) | 0.68 (0.08) | 0.83  (0.04) |
|  | **(STANDARD,**  **LUNG)** | 35.93  (6.11) | 20.97  (5.62) | 14.06 (1.41) | 18.89 (2.1) | 0.63 (0.07) | 0.79  (0.05) |
|  | **(C, D)** | 29.00  (3.50) | 27.83  (5.78) | 15.87 (1.05) | 16.35 (1.81) | 0.69 (0.05) | 0.73  (0.07) |
|  | **(B, D)** | 21.72  (4.34) | 18.72  (3.46) | 18.45 (1.43) | 19.75 (1.52) | 0.75 (0.05) | 0.80  (0.03) |
| **Subcutaneous adipose tissue** | **(B30f, B50f)** | 24.07  (5.32) | 12.64  (2.81) | 16.67 (1.94) | 22.23 (1.75) | 0.74 (0.04) | 0.91  (0.03) |
|  | **(B30f, B80f)** | 41.96  (4.80) | 12.72  (3.89) | 11.69 (1.05) | 22.31 (2.25) | 0.61 (0.04) | 0.92  (0.03) |
|  | **(STANDARD,**  **BONE)** | 28.03  (7.20) | 15.71  (4.14) | 15.43 (2.32) | 20.42 (2.08) | 0.72 (0.06) | 0.87  (0.05) |
|  | **(STANDARD,**  **LUNG)** | 31.66  (7.27) | 18.58  (5.00) | 14.30 (2.02) | 18.96 (2.04) | 0.69 (0.05) | 0.84  (0.06) |
|  | **(C, D)** | 24.66  (4.58) | 20.90  (5.08) | 16.40  (1.68) | 17.94 (2.11) | 0.76 (0.04) | 0.81  (0.05) |
|  | **(B, D)** | 16.28  (4.85) | 15.40  (2.71) | 20.14 (2.08) | 20.45 (1.41) | 0.84 (0.06) | 0.86  (0.05) |

Image similarity metrics computed for lung window ([-1000,0] Hounsfield units), subcutaneous adipose tissue window ([-190, -30] Hounsfield units) and skeletal muscle window ([-29,150] Hounsfield units) for reconstruction kernels in the NLST at timepoint T1. All metrics are expressed as the mean with standard deviation in parentheses. RMSE values decrease, PSNR and SSIM values increase post harmonization. Wilcoxon signed ranked test is used to test for significance before and after harmonization. The Benjamini-Hochberg procedure was applied to adjust for multiple comparisons and control the false discovery rate. All values except the RMSE and PSNR computed for subcutaneous adipose tissue for the pair (B, D) were significant (*p*<0.05)

**Table ST4.** **Image similarity metrics obtained for paired kernels at timepoint T2.**

| **Region of interest** | **Kernel pairs** | **RMSE** | | **PSNR** | | **SSIM** | |
| --- | --- | --- | --- | --- | --- | --- | --- |
|  |  | **Before** | **After** | **Before** | **After** | **Before** | **After** |
| **Lung** | **(B30f, B50f)** | 46.05 (10.66) | 29.10  (4.72) | 26.94 (1.81) | 30.83 (1.34) | 0.72 (0.05) | 0.93 (0.02) |
|  | **(B30f, B80f)** | 108.51 (24.70) | 28.67 (4.38) | 19.48 (1.73) | 30.93 (1.12) | 0.51 (0.03) | 0.93 (0.03) |
|  | **(STANDARD,**  **BONE)** | 55.33  (20.10) | 36.20 (5.71) | 25.64 (2.80) | 28.92 (1.30) | 0.69 (0.07) | 0.89 (0.05) |
|  | **(STANDARD,**  **LUNG)** | 75.00 (24.87) | 47.28 (8.25) | 22.91 (2.57) | 26.63 (1.47) | 0.63 (0.07) | 0.87 (0.05) |
|  | **(C, D)** | 52.25  (11.60) | 44.96 (7.83) | 25.83 (1.83) | 27.07 (1.44) | 0.73 (0.04) | 0.83 (0.05) |
|  | **(B, D)** | 37.92 (15.28) | 37.00 (4.71) | 28.93 (2.75) | 28.70 (1.07) | 0.85 (0.10) | 0.88 (0.04) |
| **Skeletal muscle** | **(B30f, B50f)** | 27.40  (4.68) | 15.07 (2.75) | 16.42 (1.45) | 21.63 (1.48) | 0.70 (0.06) | 0.87 (0.03) |
|  | **(B30f, B80f)** | 45.53  (5.42) | 15.22  (3.10) | 11.95  (1.00) | 21.56 (1.52) | 0.55 (0.06) | 0.87 (0.03) |
|  | **(STANDARD,**  **BONE)** | 32.00  (6.82) | 18.03 (4.62) | 15.15 (1.85) | 20.19 (2.08) | 0.67 (0.08) | 0.82 (0.05) |
|  | **(STANDARD,**  **LUNG)** | 36.36  (5.90) | 21.01 (4.91) | 13.96 (1.37) | 18.83 (1.91) | 0.62 (0.07) | 0.80 (0.05) |
|  | **(C, D)** | 30.35  (3.47) | 28.89 (6.02) | 15.47 (0.98) | 16.03 (1.81) | 0.68 (0.05) | 0.71 (0.07) |
|  | **(B, D)** | 23.66  (6.42) | 20.17 (4.88) | 17.84 (2.05) | 19.19 (1.93) | 0.74 (0.06) | 0.79 (0.04) |
| **Subcutaneous adipose tissue** | **(B30f, B50f)** | 24.59  (5.47) | 12.89 (2.92) | 16.48 (1.97) | 22.07 (1.79) | 0.73 (0.04) | 0.91 (0.03) |
|  | **(B30f, B80f)** | 41.77  (4.91) | 12.95 (3.76) | 11.72 (1.02) | 22.13 (2.14) | 0.62 (0.04) | 0.91 (0.03) |
|  | **(STANDARD,**  **BONE)** | 29.29  (7.60) | 16.18 (4.58) | 15.06 (2.37) | 20.3 (2.22) | 0.72 (0.06) | 0.86 (0.05) |
|  | **(STANDARD,**  **LUNG)** | 32.39  (7.15) | 18.64 (4.48) | 14.09 (1.98) | 18.89 (1.90) | 0.69 (0.04) | 0.84 (0.06) |
|  | **(C, D)** | 26.01  (4.55) | 21.67 (5.41) | 15.92 (1.57) | 17.64 (2.18) | 0.75 (0.03) | 0.80 (0.05) |
|  | **(B, D)** | 18.27  (6.91) | 16.29 (3.57) | 19.34 (2.77) | 20.03 (1.76) | 0.82 (0.07) | 0.85 (0.05) |

Image similarity metrics computed for lung window ([-1000,0] Hounsfield units), subcutaneous adipose tissue window ([-190, -30] Hounsfield units) and skeletal muscle window ([-29,150] Hounsfield units) for reconstruction kernels in the NLST at timepoint T2. All metrics are expressed as the mean with standard deviation in parentheses. RMSE values decrease, PSNR and SSIM values increase post harmonization. Wilcoxon signed ranked test is used to test for significance before and after harmonization. The Benjamini-Hochberg procedure was applied to adjust for multiple comparisons and control the false discovery rate. All pairs were statistically significant (*p* < 0.05).

**Table ST5: Comparison of image similarity metrics between histogram matching (baseline) and multipath cycleGAN (proposed) at timepoint T0 for the lung**

| **Kernel pairs** | **RMSE** | | | **PSNR** | | | **SSIM** | | |
| --- | --- | --- | --- | --- | --- | --- | --- | --- | --- |
|  | **Before** | **After (proposed)** | **After (baseline)** | **Before** | **After**  **(proposed)** | **After (baseline)** | **Before** | **After (proposed)** | **After (baseline)** |
| **(B30f, B50f)** | 43.31 (10.06) | 27.66 (4.21) | 38.71 (6.77) | 27.45 (1.73) | 31.25 (1.22) | 28.35 (1.27) | 0.73 (0.05) | 0.94  (0.02) | 0.77 (0.04) |
| **(B30f, B80f)** | 94.41 (22.02) | 27.55 (3.17) | 81.91 (19.12) | 20.70  (1.80) | 31.25 (0.91) | 21.92 (1.72) | 0.53 (0.04) | 0.94  (0.02) | 0.60 (0.04) |
| **(STANDARD,**  **BONE)** | 56.23 (17.12) | 37.09 (5.57) | 47.68 (12.58) | 25.36 (2.47) | 28.71 (1.26) | 26.70  (2.09) | 0.68 (0.06) | 0.88  (0.05) | 0.73 (0.05) |
| **(STANDARD,**  **LUNG)** | 75.22 (29.32) | 47.15 (7.19) | 66.79 (24.44) | 23.00  (2.89) | 26.62 (1.23) | 23.94 (2.58) | 0.63 (0.08) | 0.87  (0.06) | 0.68 (0.06) |
| **(C, D)** | 37.82  (9.58) | 36.98 (6.78) | 33.89  (7.76) | 28.69 (1.97) | 28.77 (1.47) | 29.60 (1.84) | 0.83 (0.09) | 0.87  (0.05) | 0.84 (0.07) |
| **(B, D)** | 39.14  (11.6) | 37.20  (6.25) | 35.27 (9.90) | 28.48 (2.33) | 28.69 (1.25) | 29.36 (2.24) | 0.83 (0.10) | 0.87  (0.05) | 0.84 (0.08) |

Comparison of image similarity metrics computed on lung window ([-1000,0] Hounsfield units)) for reconstruction kernels in the NLST at timepoint T0 between our proposed approach and a simple histogram matching baseline. All metrics are expressed as the mean with standard deviation in parentheses. RMSE values decrease, PSNR and SSIM values increase post harmonization.

**Table ST6: Comparison of image similarity metrics between histogram matching (baseline) and multipath cycleGAN (proposed) at timepoint T0 for skeletal muscle**

| **Kernel pairs** | **RMSE** | | | **PSNR** | | | **SSIM** | | |
| --- | --- | --- | --- | --- | --- | --- | --- | --- | --- |
|  | **Before** | **After**  **(proposed)** | **After (baseline)** | **Before** | **After**  **(proposed)** | **After (baseline)** | **Before** | **After**  **(proposed)** | **After (baseline)** |
| **(B30f, B50f)** | 26.39  (4.43) | 14.47 (2.73) | 21.48 (3.39) | 16.74 (1.40) | 21.98 (1.47) | 18.52 (1.31) | 0.71 (0.06) | 0.87  (0.03) | 0.74 (0.05) |
| **(B30f, B80f)** | 42.56  (5.18) | 14.14 (2.53) | 31.40 (2.95) | 12.54 (1.02) | 22.17 (1.43) | 15.16 (0.80) | 0.57 (0.07) | 0.88  (0.03) | 0.63 (0.07) |
| **(STANDARD,**  **BONE)** | 32.19  (6.00) | 18.85 (4.61) | 26.45 (4.76) | 15.05 (1.61) | 19.79 (2.03) | 16.75 (1.55) | 0.67 (0.07) | 0.82  (0.04) | 0.71 (0.06) |
| **(STANDARD,**  **LUNG)** | 36.29  (6.60) | 21.00  (5.82) | 27.82 (4.06) | 14.00  (1.53) | 18.90 (2.15) | 16.26 (1.22) | 0.62 (0.07) | 0.79  (0.06) | 0.67 (0.06) |
| **(C, D)** | 24.25  (4.10) | 23.32 (5.34) | 18.27 (3.38) | 17.48 (1.40) | 17.91 (1.97) | 19.96 (1.54) | 0.73 (0.06) | 0.77  (0.06) | 0.79 (0.05) |
| **(B, D)** | 24.83  (5.37) | 20.28  (4.80) | 18.97 (4.16) | 17.35 (1.82) | 19.15 (2.01) | 19.68 (1.78) | 0.72 (0.07) | 0.78  (0.05) | 0.78 (0.06) |

Comparison of image similarity metrics computed on skeletal window ([-29,150] Hounsfield units)) for reconstruction kernels in the NLST at timepoint T0 between our proposed approach and a simple histogram matching baseline. All metrics are expressed as the mean with standard deviation in parentheses. RMSE values decrease, PSNR and SSIM values increase post harmonization.

**Table ST7: Comparison of image similarity metrics between histogram matching (baseline) and multipath cycleGAN (proposed) at timepoint T0 for subcutaneous adipose tissue**

| **Kernel pairs** | **RMSE** | | | **PSNR** | | | **SSIM** | | |
| --- | --- | --- | --- | --- | --- | --- | --- | --- | --- |
|  | **Before** | **After**  **(proposed)** | **After (baseline)** | **Before** | **After (proposed)** | **After (baseline)** | **Before** | **After (proposed)** | **After (baseline)** |
| **(B30f, B50f)** | 23.37  (5.28) | 12.39 (2.79) | 19.25 (3.65) | 16.93 (1.98) | 22.40 (1.69) | 18.55 (1.63) | 0.74 (0.04) | 0.92  (0.03) | 0.76 (0.03) |
| **(B30f, B80f)** | 38.49  (5.84) | 11.17 (3.06) | 27.94 (4.05) | 12.48 (1.34) | 23.38 (1.99) | 15.25 (1.26) | 0.61 (0.04) | 0.93  (0.03) | 0.64 (0.04) |
| **(STANDARD,**  **BONE)** | 29.39  (6.65) | 16.82  (4.60) | 23.70 (4.85) | 14.95 (2.03) | 19.85 (2.17) | 16.77 (1.79) | 0.72 (0.05) | 0.85  (0.05) | 0.74 (0.05) |
| **(STANDARD,**  **LUNG)** | 31.63  (7.88) | 18.71 (5.24) | 24.43 (4.69) | 14.36 (2.21) | 18.92 (2.08) | 16.48 (1.63) | 0.69 (0.05) | 0.84  (0.07) | 0.72 (0.04) |
| **(C, D)** | 19.12  (5.43) | 18.02 (4.79) | 16.39 (3.69) | 18.78 (2.37) | 19.25 (2.20) | 20.00 (1.86) | 0.82 (0.06) | 0.84  (0.05) | 0.83 (0.05) |
| **(B, D)** | 19.53  (6.37) | 16.55 (3.94) | 16.92 (4.32) | 18.69 (2.65) | 19.94 (1.99) | 19.78 (2.11) | 0.80 (0.08) | 0.84  (0.07) | 0.81 (0.06) |

Comparison of image similarity metrics computed on subcutaneous adipose tissue window ([-190,-30] Hounsfield units)) for reconstruction kernels in the NLST at timepoint T0 between our proposed approach and a simple histogram matching baseline. All metrics are expressed as the mean with standard deviation in parentheses. RMSE values decrease, PSNR and SSIM values increase post harmonization.

**Table ST8. Emphysema and body composition measures for paired kernels at timepoint T1.**

| **Kernel pairs** | **Emphysema (%)** | | **Skeletal muscle (cm^3^)** | | **Subcutaneous adipose tissue (cm^3^)** | |
| --- | --- | --- | --- | --- | --- | --- |
|  | **Before** | **After** | **Before** | **After** | **Before** | **After** |
| **(B30f,**  **B50f)** | 11.77 [11.69, 11.84] | 1.61  [1.58, 1.65] | 98.64  [95.24, 103.72] | 46.81  [44.95, 49.99] | 245.64 [230.36, 268.17] | 58.38 [56.01, 62.11] |
| **(B30f,**  **B80f)** | 28.87 [27.95, 29.71] | 2.64  [2.34, 2.98] | 274.48 [171.05, 478.36] | 92.16  [68.78, 128.13] | 885.74 [638.56, 1235.06] | 114.05 [90.24, 144.6] |
| **(STANDARD, BONE)** | 8.71 [8.59, 8.82] | 0.93  [0.88, 0.98] | 149.75 [144.89, 159.52] | 50.16  [48.30, 52.53] | 434.91 [417.54, 459.27] | 83.00 [78.68, 89.59] |
| **(STANDARD, LUNG)** | 19.53 [19.23, 19.82] | 1.45  [1.29, 1.64] | 227.52 [200.15, 276.63] | 56.60  [50.45, 69.65] | 798.13 [718.13, 923.55] | 78.14 [68.57, 98.26] |
| **(C, D)** | 14.26 [14.00, 14.50] | 4.96  [4.76, 5.13] | 206.37 [196.47, 216.3] | 141.52 [133.74, 151.47] | 383.26 [368.30, 405.29] | 147.96 [135.58, 167.49] |
| **(B, D)** | 12.26 [11.43, 13.37] | 1.22  [1.02, 1.52] | 123.17 [103.49, 150.95] | 97.69 [83.69,115.34] | 250.08 [223.42, 286.16] | 119.41 [103.78, 139.1] |

Percent emphysema, skeletal muscle volume and subcutaneous adipose tissue volumes computed for reconstruction kernel pairs at timepoint T1. The measures are expressed as the root mean square error (RMSE) with 95% confidence intervals in square brackets. RMSE values decrease post harmonization for all pairs. Statistical significance for all measurements before and after harmonization were determined by a Wilcoxon signed rank test with bootstrapping (*p*<0.05). The Benjamini-Hochberg procedure was applied to adjust for multiple comparisons and control the false discovery rate. For emphysema and subcutaneous adipose tissue, all pairs were statistically significant. For skeletal muscle, all pairs except for (B, D) were significant.

**Table ST9. Emphysema and body composition measures obtained for paired kernels at timepoint T2.**

| **Kernel pairs** | **Emphysema (%)** | | **Skeletal muscle (cm^3^)** | | **Subcutaneous adipose tissue (cm^3^)** | |
| --- | --- | --- | --- | --- | --- | --- |
|  | **Before** | **After** | **Before** | **After** | **Before** | **After** |
| **(B30f,**  **B50f)** | 11.91 [11.83, 11.98] | 1.66  [1.61, 1.70] | 97.94  [94.29, 103.90] | 45.19  [43.45, 48.39 | 256.62 [240.67, 283.33] | 61.52 [58.79, 65.63] |
| **(B30f,**  **B80f)** | 27.74 [25.90, 29.02] | 2.62  [2.30, 3.04] | 361.01 [137.05, 734.96] | 58.87  [44.51, 81.20] | 952.52 [491.22, 1598.13] | 92.36 [63.25, 147.43] |
| **(STANDARD, BONE)** | 9.64  [9.52, 9.77] | 1.09  [1.04, 1.17] | 162.83 [157.28, 171.08] | 57.73  [53.58, 67.29] | 493.48 [472.11, 516.94] | 97.54 [86.98, 136.39] |
| **(STANDARD, LUNG)** | 19.83 [19.51, 20.16] | 1.52  [1.36, 1.76] | 247.75 [218.95, 291.86] | 56.28  [49.16, 66.64] | 889.43 [794.49, 1016.38] | 91.23 [78.22, 111.90] |
| **(C, D)** | 14.63 [14.37, 14.88] | 5.52  [5.31, 5.70] | 212.11 [200.99, 225.14] | 166.43 [156.93, 176.03] | 376.97 [360.43, 397.45] | 136.11 [124.32, 153.74] |
| **(B, D)** | 13.59 [12.66, 14.51] | 1.22  [1.04, 1.47] | 171.11  [ 144.27, 209.17] | 130.90 [112.28, 159.63] | 295.78 [262.12, 342.86] | 141.83 [125.21, 169.56] |

Percent emphysema, skeletal muscle volume and subcutaneous adipose tissue volumes computed for reconstruction kernel pairs at timepoint T2. The measures are expressed as the root mean square error (RMSE) with 95% confidence intervals in square brackets. RMSE values decrease post harmonization for all pairs. Statistical significance for all measurements before and after harmonization were determined by a Wilcoxon signed rank test with bootstrapping (*p*<0.05). The Benjamini-Hochberg procedure was applied to adjust for multiple comparisons and control the false discovery rate. For emphysema and subcutaneous adipose tissue, all pairs were statistically significant. For skeletal muscle, all pairs except for (B, D) were significant.

**Table ST10. Cohen’s *d* effect sizes obtained on matched samples at timepoint T1.**

| **Reference kernel** | **Reconstruction kernel** | **Cohen’s d before harmonization** | **Cohen’s d after harmonization** |
| --- | --- | --- | --- |
| **Siemens B30f** | **Siemens B50f** | -1.51 | -0.1 |
|  | **Siemens B80f** | -3.92 | -0.21 |
|  | **GE BONE** | -0.33 | 0.14 |
|  | **GE STANDARD** | 0.81 | -0.25 |
|  | **GE LUNG** | -1.61 | 0.01 |
|  | **Philips B** | 1.08 | 0.68 |
|  | **Philips C** | 0.93 | 0.43 |
|  | **Philips D** | -0.99 | -0.06 |
| **Siemens B50f** | **Siemens B30f** | 1.51 | 0.24 |
|  | **Siemens B80f** | -2.48 | -0.30 |
|  | **GE BONE** | 1.38 | 0.38 |
|  | **GE STANDARD** | 2.80 | 0.25 |
|  | **GE LUNG** | -0.13 | 0.19 |
|  | **Philips B** | 3.13 | 0.94 |
|  | **Philips C** | 3.08 | 0.25 |
|  | **Philips D** | 0.67 | 0.41 |

We present Cohen’s *d* effect sizes computed between the emphysema distributions of the reference kernel and source kernel before and after harmonization for matched samples at timepoint T1. Cohen’s *d* quantifies the magnitude of difference between the emphysema scores, highlighting kernel bias. Effect sizes are small when *d* = 0.2, medium when *d*=0.5 and large when *d*≥0.8. A positive *d* indicates that group one had a higher mean compared to group two while a negative *d* indicates the opposite. Harmonization to the reference reduces bias in emphysema measures as evidenced by a shift from large effect sizes to small or medium effect sizes across different kernels.

**Table ST11.** **Cohen’s *d* effect sizes obtained on matched samples at timepoint T2.**

| **Reference kernel** | **Reconstruction kernel** | **Cohen’s d before harmonization** | **Cohen’s d after harmonization** |
| --- | --- | --- | --- |
| **Siemens B30f** | **Siemens B50f** | -1.85 | -0.31 |
|  | **Siemens B80f** | -4.03 | -0.53 |
|  | **GE BONE** | -0.84 | -0.37 |
|  | **GE STANDARD** | 0.63 | -0.33 |
|  | **GE LUNG** | -1.73 | -0.06 |
|  | **Philips B** | 0.51 | 0.09 |
|  | **Philips C** | 0.61 | 0.19 |
|  | **Philips D** | -1.18 | -0.36 |
| **Siemens B50f** | **Siemens B30f** | 1.85 | 0.48 |
|  | **Siemens B80f** | -2.42 | -0.32 |
|  | **GE BONE** | 1.01 | 0.10 |
|  | **GE STANDARD** | 3.0 | 0.42 |
|  | **GE LUNG** | 0.00 | 0.44 |
|  | **Philips B** | 2.6 | 0.54 |
|  | **Philips C** | 3.01 | 0.39 |
|  | **Philips D** | 0.55 | 0.41 |

We present Cohen’s *d* effect sizes computed between the emphysema distributions of the reference kernel and source kernel before and after harmonization for matched samples at timepoint T2. Cohen’s *d* quantifies the magnitude of difference between the emphysema scores, highlighting kernel bias. Effect sizes are small when *d* = 0.2, medium when *d*=0.5 and large when *d*≥0.8. A positive *d* indicates that group one had a higher mean compared to group two while a negative *d* indicates the opposite. Harmonization to the reference reduces bias in emphysema measures as evidenced by a shift from large effect sizes to small or medium effect sizes across different kernels.

**Table ST12. Body composition correlations post harmonization to Siemens B30f and FOV extension at timepoint T1.**

| **Kernel** | **SAT index (cm^3^/m^2^) vs FM index (kg/m^2^)** | | | **Muscle index (cm^3^/m^2^) vs FFM index (kg/m^2^)** | | |
| --- | --- | --- | --- | --- | --- | --- |
|  | **Truncated** | **FOV extended** | ***p*-value** | **Truncated** | **FOV extended** | ***p*-value** |
| **Siemens B30f** | 0.814  [0.805,0.823] | 0.828  [0.820,0.837] | <0.05 | 0.729  [0.718,0.74] | 0.729  [0.718,0.739] | 0.82 |
| **Siemens B50f** | 0.799  [0.788,0.81] | 0.832  [0.823,0.842] | <0.05 | 0.717  [0.704,0.729] | 0.733  [0.721,0.745] | <0.05 |
| **Siemens B80f** | 0.831  [0.753,0.885] | 0.865  [0.796,0.907] | <0.05 | 0.782  [0.694,0.847] | 0.774  [0.679,0.836] | 0.35 |
| **GE BONE** | 0.707  [0.692,0.720] | 0.829  [0.817,0.839] | <0.05 | 0.686  [0.672,0.701] | 0.704  [0.689,0.717] | <0.05 |
| **GE STANDARD** | 0.751  [0.742,0.760] | 0.822  [0.815,0.829] | <0.05 | 0.664  [0.654,0.674] | 0.718  [0.709,0.727] | <0.05 |
| **GE LUNG** | 0.725  [0.697,0.751] | 0.829  [0.807,0.848] | <0.05 | 0.684  [0.654,0.712] | 0.707  [0.680,0.731] | <0.05 |
| **Philips B** | 0.806  [0.711,0.864] | 0.847  [0.769,0.895] | <0.05 | 0.638  [0.497,0.744] | 0.659  [0.522,0.761] | 0.31 |
| **Philips C** | 0.785  [0.761,0.807] | 0.813  [0.792,0.833] | <0.05 | 0.708  [0.679,0.734] | 0.711  [0.683,0.736] | 0.21 |
| **Philips D** | 0.783  [0.749,0.811] | 0.822  [0.794,0.846] | <0.05 | 0.670  [0.625,0.709] | 0.687  [0.646,0.725] | <0.05 |

Comparison of body composition correlations between the body composition volume indices normalized by square of the height and the approximated anthropometric body composition indices before and after FOV extension at timepoint T1. FOV extension is performed on the images harmonized to the reference Siemens B30f kernel. The approximations were derived from the height and weight of the subject at baseline. We report the spearman rank correlations with 95% confidence intervals. Significance before and after FOV extension was evaluated using the method implemented by Hittner et al. FM = Fat mass, FFM = Fat free mass, SAT = Subcutaneous adipose tissue.

**Table ST13. Body composition correlations post harmonization to Siemens B30f and FOV extension at timepoint T2.**

| **Kernel** | **SAT index (cm^3^/m^2^) vs FM index (kg/m^2^)** | | | **Muscle index (cm^3^/m^2^) vs FFM index (kg/m^2^)** | | |
| --- | --- | --- | --- | --- | --- | --- |
|  | **Truncated** | **FOV extended** | ***p*-value** | **Truncated** | **FOV extended** | ***p*-value** |
| **Siemens B30f** | 0.802  [0.793,0.812] | 0.816  [0.807,0.825] | <0.05 | 0.710  [0.699,0.721] | 0.712  [0.701,0.723] | 0.05 |
| **Siemens B50f** | 0.788  [0.776,0.799] | 0.817  [0.806,0.826] | <0.05 | 0.710  [0.695,0.724] | 0.723  [0.709,0.736] | <0.05 |
| **Siemens B80f** | 0.845  [0.731,0.906] | 0.855  [0.745,0.913] | 0.09 | 0.665  [0.491,0.78] | 0.6  [0.402,0.749] | <0.05 |
| **GE BONE** | 0.679 [0.663,0.695] | 0.811  [0.8,0.821] | <0.05 | 0.72  [0.709,0.731] | 0.72  [0.709,0.732] | 0.9 |
| **GE STANDARD** | 0.741  [0.732,0.749] | 0.811  [0.804,0.819] | <0.05 | 0.658  [0.647,0.668] | 0.711  [0.702,0.72] | <0.05 |
| **GE LUNG** | 0.698  [0.664,0.727] | 0.82  [0.796,0.841] | <0.05 | 0.691  [0.658,0.718] | 0.709  [0.681,0.734] | <0.05 |
| **Philips B** | 0.788  [0.717,0.846] | 0.821  [0.752,0.871] | <0.05 | 0.606  [0.482,0.71] | 0.648  [0.537,0.738] | 0.04 |
| **Philips C** | 0.764  [0.736,0.788] | 0.795  [0.768,0.817] | <0.05 | 0.702  [0.673,0.73] | 0.715  [0.687,0.743] | <0.05 |
| **Philips D** | 0.755  [0.72,0.787] | 0.811  [0.784,0.836] | <0.05 | 0.664  [0.618,0.704] | 0.676  [0.628,0.716] | 0.02 |

Comparison of body composition correlations between the body composition volume indices normalized by square of the height and the approximated anthropometric body composition indices before and after FOV extension at timepoint T2. FOV extension is performed on the images harmonized to the reference Siemens B30f kernel. The approximations were derived from the height and weight of the subject at baseline. We report the spearman rank correlations with 95% confidence intervals. Significance before and after FOV extension was evaluated using the method implemented by Hittner et al. FM = Fat mass, FFM = Fat free mass, SAT = Subcutaneous adipose tissue.

**Table ST14. Body composition correlations post harmonization to Siemens B50f and FOV extension at timepoint T1.**

| **Kernel** | **SAT index (cm^3^/m^2^) vs FM index (kg/m^2^)** | | | **Muscle index (cm^3^/m^2^) vs FFM index (kg/m^2^)** | | |
| --- | --- | --- | --- | --- | --- | --- |
|  | **Truncated** | **FOV extended** | ***p*-value** | **Truncated** | **FOV extended** | ***p*-value** |
| **Siemens B30f** | 0.814  [0.805,0.823] | 0.822  [0.814,0.831] | <0.05 | 0.729  [0.719,0.74] | 0.734  [0.723,0.744] | <0.05 |
| **Siemens B50f** | 0.799  [0.787,0.809] | 0.821  [0.81,0.83] | <0.05 | 0.717  [0.704,0.729 | 0.72  [0.708,0.732] | 0.01 |
| **Siemens B80f** | 0.831  [0.753,0.885] | 0.858  [0.788,0.901] | <0.05 | 0.782  [0.694,0.847] | 0.789  [0.695,0.849] | 0.36 |
| **GE BONE** | 0.707  [0.692,0.721] | 0.823  [0.812,0.833] | <0.05 | 0.687  [0.672,0.701] | 0.718  [0.705,0.731] | <0.05 |
| **GE STANDARD** | 0.751  [0.742,0.76] | 0.815  [0.808,0.822] | <0.05 | 0.664  [0.654,0.674] | 0.721  [0.713,0.73] | <0.05 |
| **GE LUNG** | 0.727  [0.701,0.754] | 0.826  [0.806,0.846] | <0.05 | 0.685  [0.655,0.715] | 0.705  [0.678,0.732] | <0.05 |
| **Philips B** | 0.81  [0.713,0.873] | 0.845  [0.763,0.895] | <0.05 | 0.64  [0.511,0.741] | 0.64  [0.498,0.754] | 0.96 |
| **Philips C** | 0.783  [0.76,0.805] | 0.802  [0.781,0.823] | <0.05 | 0.709  [0.68,0.734] | 0.698  [0.668,0.724] | <0.05 |
| **Philips D** | 0.8  [0.771,0.824] | 0.828  [0.803,0.851] | <0.05 | 0.663  [0.619,0.704] | 0.677  [0.63,0.716] | <0.05 |

Comparison of body composition correlations between the body composition volume indices normalized by square of the height and the approximated anthropometric body composition indices before and after FOV extension at timepoint T1. FOV extension is performed on the images harmonized to the reference Siemens B50f kernel. The approximations were derived from the height and weight of the subject at baseline. We report the spearman rank correlations with 95% confidence intervals. Significance before and after FOV extension was evaluated using the method implemented by Hittner et al. FM = Fat mass, FFM = Fat free mass, SAT = Subcutaneous adipose tissue.

**Table ST15. Body composition correlations post harmonization to Siemens B50f and FOV extension at timepoint T2.**

| **Kernel** | **SAT index (cm^3^/m^2^) vs FM index (kg/m^2^)** | | | **Muscle index (cm^3^/m^2^) vs FFM index (kg/m^2^)** | | |
| --- | --- | --- | --- | --- | --- | --- |
|  | **Truncated** | **FOV extended** | ***p*-value** | **Truncated** | **FOV extended** | ***p*-value** |
| **Siemens B30f** | 0.802  [0.792,0.812] | 0.81  [0.801,0.82] | <0.05 | 0.71  [0.698,0.721] | 0.72  [0.707,0.73] | <0.05 |
| **Siemens B50f** | 0.788  [0.777,0.8] | 0.806  [0.796,0.817] | <0.05 | 0.71  [0.696,0.723] | 0.709  [0.694,0.723] | 0.47 |
| **Siemens B80f** | 0.845  [0.731,0.906] | 0.851  [0.741,0.911] | 0.28 | 0.665  [0.491,0.78] | 0.653  [0.473,0.78] | 0.41 |
| **GE BONE** | 0.68  [0.664,0.695] | 0.805  [0.794,0.815] | <0.05 | 0.72  [0.708,0.73] | 0.737  [0.726,0.748] | <0.05 |
| **GE STANDARD** | 0.741  [0.732,0.75] | 0.804  [0.796,0.811] | <0.05 | 0.658  [0.647,0.668] | 0.717  [0.708,0.726] | <0.05 |
| **GE LUNG** | 0.698  [0.667,0.729] | 0.814  [0.791,0.836] | <0.05 | 0.691  [0.66,0.718] | 0.71  [0.681,0.736] | <0.05 |
| **Philips B** | 0.789  [0.711,0.852] | 0.815  [0.743,0.87] | <0.05 | 0.605  [0.47,0.705] | 0.624  [0.491,0.718] | 0.24 |
| **Philips C** | 0.762  [0.733,0.787] | 0.784  [0.758,0.808] | <0.05 | 0.697  [0.669,0.726] | 0.699  [0.668,0.727] | 0.73 |
| **Philips D** | 0.764  [0.73,0.792] | 0.805  [0.776,0.829] | <0.05 | 0.659  [0.617,0.695] | 0.669  [0.629,0.704] | 0.04 |

Comparison of body composition correlations between the body composition volume indices normalized by square of the height and the approximated anthropometric body composition indices before and after FOV extension at timepoint T2. FOV extension is performed on the images harmonized to the reference Siemens B50f kernel. The approximations were derived from the height and weight of the subject at baseline. We report the spearman rank correlations with 95% confidence intervals. Significance before and after FOV extension was evaluated using the method implemented by Hittner et al. FM = Fat mass, FFM = Fat free mass, SAT = Subcutaneous adipose tissue.
